# *In silico* assessment of CAR macrophages activity against SARS-CoV-2 infection

**DOI:** 10.1016/j.heliyon.2024.e39689

**Published:** 2024-10-23

**Authors:** Antonino Amoddeo

**Affiliations:** Department of Civil, Energy, Environment and Materials Engineering, Università’Mediterranea’ di Reggio Calabria, Via R. Zehender 1, Feo di Vito, I-89122, Reggio Calabria, Italy

## Abstract

Macrophage engineering with chimeric antigen receptor is a promising technique first applied to the treatment of tumours and recently suggested as a possible immunotherapeutic route against the COVID-19 disease. Four immunotherapies based on engineered macrophages have been tested *in vitro* revealing promising, with one of them acting without increasing the cytokines level. We present a mathematical model aimed at the evaluation of both the SARS-CoV-2 virions dynamics and the cytokines production induced, while such newly developed constructs interact with the immune system once administered. The importance of the study lies both in monitoring the dynamics of the infection and in evaluating the cytokine production, since clinical studies show that in critical COVID-19 patients an abnormal cytokines production occurs, a concern to be accounted for in designing appropriate therapeutic strategies. The mathematical model was built in the context of the continuum approach of the mass conservation, while the numerical simulations have been performed introducing parameters deduced from the experiments, using the finite element method. The model simulations allow to analyse and to compare the immune mechanisms underlying the virus dynamics, deepening the investigation for two selected immunotherapies, suggesting that a synergistic work of involved cytokines with phagocytic activity of macrophages occurs. The best SARS-CoV-2 clearance relies not only on the phagocytic capacity of the engineered macrophages, but also on the production of T-lymphocytes, pro- and anti-inflammatory cytokines which in the two cases examined in depth can decrease by 99.7 %, 99.6 % and 69 % respectively, passing from the most effective immunotherapy to the least effective one. This study is the first mathematical model that analyses the dynamics of macrophages engineered to fight the COVID-19, and paves the way for their possible exploitation against such a challenging disease, going beyond existing models involving other immune cells.

## 1 Introduction

The innate immune system provides a first defence line against threatening pathogens intruding the human body and includes, among others, natural killer (NK) cells and macrophages (M). These last can be grouped into classically activated macrophages (M1), and alternatively activated macrophages (M2), having distinct functions [[1], [2], [3], [4], [5], [6]]. Interleukins (ILs) are cytokines which regulate the cellular activity triggering molecular signaling, and are commonly divided into pro- and anti-inflammatory ILs, but pleiotropic effects are possible [[7], [8], [9], [10]]. M1 promote T lymphocytes, in particular the CD4^+^ T-cells, or T-helper (T_h_) cells, and ultimately induce the production of inflammatory interleukins (ILs) as IL-6, and interferon-γ (IFN-γ) [5], with the activation of more CD4^+^ T-cells, then a pro-inflammatory role is attributed to them. M2, instead, have a restoration and healing function [[1], [2], [3], [4],^11^], and are considered as anti-inflammatory agents since they trigger the production of anti-inflammatory interleukins as IL-10 [6]. For more insights we refer interested readers, among others, to papers [1,[12], [13], [14]] and references therein.

Equipping specific immune cells with chimeric antigen receptors (CARs) is a promising technique that is developing alongside research efforts for cancer immunotherapies [[15], [16], [17]]. The CAR technology was introduced in the late 80s, early 90s [18,19], and optimized over the years with the aim of strengthening T cells and allowing their widespread use. CARs are synthetic receptors originally intended to reprogram T lymphocytes to target chosen antigens, and are constituted by an extracellular domain, a transmembrane domain and an intracellular and signaling domain [16,17]. The extracellular domain comprises a target (antigen) binding domain having the scope to bind a specific antigen located on the surface of a pathogen, and a hinge or spacer domain having essentially a mechanical function; the transmembrane domain docks the CAR to the immune cell surface, while the intracellular and signaling domain provides activation of the immune cell.

The use of engineered T cells with CARs (CAR-T cells) has been authorized by the US Food and Drug Administration (FDA) for the treatment of selected onco-haematic diseases [17], while equally promising immunotherapies based on CAR engineered NK (CAR-NK) cells [16,20] are appearing, even if there are recent news that the FDA has been informed about T-cells malignancies emerging in patients treated with CAR-T immunotherapies, and on this basis, ‘although the overall benefits of these products continue to outweigh their potential risks for their approved uses, FDA is investigating the identified risk of T cell malignancy with serious outcomes, including hospitalization and death, and is evaluating the need for regulatory action’ [21].

Nevertheless, CAR-T therapies fail in the treatment of solid malignancies due to the resistance opposed by the tumour microenvironment (TME), while CAR-NK therapies, having a short half-life, require repeated administration in clinical treatments [16].

Vincent et al. [22] reported on a new developed platform of probiotic-guided CAR-T cells, allowing tumour tissue to be labeled by a specific antigen released by probiotics that colonize the tumour, and later attacked by CAR-T programmed to recognize them.

Exploiting their ability to overcome the barrier opposed by solid tumours, macrophages engineered with CARs (CAR-M) have been recently constructed as a new therapeutic tool against solid tumours [15,16,[23], [24], [25], [26]]. In addition to having the ability to trigger the immune system response, macrophages perform phagocytic activity against pathogens: a comparison among the efficacy of the immunotherapies designed for cancer treatment based on the above three CARs has been presented in the review of Pan et al. [16] where for each therapy advantages and limitations are analysed.

In Liu et al. [27] the synergistic effect of combined CAR-T/CAR-M therapy to fight cancer is investigated, evidencing as CAR-T cells induce augmented cytotoxicity of CAR-M and M2 polarization towards the M1 phenotype.

The outbreak of the severe acute respiratory syndrome corona virus 2 (SARS-CoV-2), also known as Corona Virus Disease - 2019 (COVID-19), is triggering new therapeutic challenges against such burdensome disease and in preparation for a possible next pandemic or other viral threats [[28], [29], [30]]. At the present time pathologists are facing the SARS-CoV-2 Omicron BA.5 variant which is causing concern as in experiments on infected aged mice a high lethality was found [31], while the newest Omicron subvariant BA.2.86 is under surveillance because of its worldwide spread [32]. In the route of immunotherapy, researchers are studying NK cells and T cells engineered with CARs to fight the COVID-19 [20,33], but one serious and general issue connected to the use of CAR-T or CAR-NK is the inflammation promotion with consequent abnormal cytokines release, the so called cytokine storm (CS) [13,14,34] that, in COVID-19 patients with an already compromised frame of inflammation, represents a possible lethal complication [14,35,36].

Recently we have modeled the SARS-CoV-2 dynamics during its interaction with the innate immune system in the early stage of the infection [12] and we have deduced as the inflammatory response is unable to significatively influence the disease progression which remains characterized by an abnormal production of ILs, markedly IL-6 but even more IL-10, as reported from early clinical observations [35,36]. While IL-6 is commonly classified as a pro-inflammatory interleukin [13,14], IL-10 may have a pleiotropic behavior [9,10], so the direct correlation between its production and the disease progression supports a pro-inflammatory role of IL-10 in the context of the COVID-19 disease [9,12,37].

In a recent work Fu et al. [38] genetically armed human macrophages with CARs to reprogram their phagocytic activity against SARS-CoV-2 based on recognition of the spike protein. These Authors conducted *in vitro* experiments on four CAR-Ms, which differ in the intracellular and signaling domain characterizing each CAR construct, labeled as CAR_γ_, CAR_MGF10_, CAR_MERTK_, CAR_ζ_, assessing for each the induced phagocytic capacity and the production of several cytokines such as IFN-γ, IL-1β, IL-2, IL-4, IL-5, IL-6, IL-8, IL-10 and tumour necrosis factor (TNF)-α. Their results demonstrate that all four CAR constructs provide macrophages with considerable phagocytic activity against SARS-CoV-2 virions, but three of these show the ability to induce significant production of ILs which, if associated with an inflammatory state in critical patients, can lead to lethal outcomes. They conclude that among the four studied CAR-Ms, CAR_MERTK_ is the one that has proven to have the highest capacity to eliminate the SARS-CoV-2 virions together with the lowest production of ILs, thus identifying it as a powerful therapy against COVID-19. Therefore, starting from their experimental results we build a mathematical model using the continuum approach with the aim to assess *in silico* the efficacy of the CAR constructs against the COVID-19, and at the same time to predict the disease evolution in patients treated with CAR-M over a period of several days, using as baseline the model presented in Ref. [12]. The present model, then, considers the mechanisms induced in COVID-19 patients upon CAR-Ms administration, specifically the increase in both phagocytic activity and cytokine production, moreover, introducing the dynamics of IFN-γ due to the pro-inflammatory bias such cytokine gives to the immune response. We consider explicitly the interaction of SARS-CoV-2 virions with M1 macrophages, healthy and infected CD4^+^ T-cells, while the immune machinery includes also M2 macrophages, IL-6, IL-10 and IFN-γ. For our purposes administered CAR-Ms have been considered as indistinguishable from the M1 phenotype. We assume that SARS-CoV-2 virions infect CD4^+^ T-cells based on some similarity with human immunodeficiency virus (HIV) - 1: HIV-1 infects CD4^+^ T-cells [39,40], while in Shen et al. [41] clinical evidences of infected T cells in peripheral blood cells (PBCs) or postmortem lung T cells of COVID-19 patients, are found, with *in vitro* experiments proving that CD4^+^ T-cells are preferably infected. We introduce a model equation for each of the above constituents accounting for their density evolution, obtaining a system of eight coupled partial differential equations (PDEs). The dynamical evolution of the system has been monitored by numerical simulations, extracting the specific parameters for each of the simulated CAR constructs from the experimental results reported in Ref. [38]. The computational results suggest that CAR-M virus clearance is not based solely on their phagocytic capacity, but the interaction among SARS-CoV-2 virions, CAR-Ms and the immune agents during a time interval of several days reveals that in the case of CAR_γ_ the virions clearance is improved with respect to CAR_MERTK_ without increasing the load of inflammatory IL-6.

## 2 Theoretical model

In the physical domain *Ω* ⊂ ℝ^3^ having *Σ* as bounding surface we monitor the space-time evolution of eight variables representing the densities of as many species: SARS-CoV-2 virions (*V*), healthy CD4^+^ T-cells (*T*), infected CD4^+^ T-cells (*I*), classically activated macrophages (*M1*), alternatively activated macrophages (*M2*), IL-6 (*L*), IL-10 (*N*) and IFN-γ (*IG*). We refer to the independent variables time *t* ∈ [0,*T*] and position ***x*** = (*x*,*y,z*) ∈ *Ω*, while the dependent variables are the concentrations of the species *s*_*i*_ = *s*_*i*_ (***x***,*t*), for *i* = 1 … 8. Introducing the vector quantity ***s*** = (*s*_*1*_ … *s*_*n*_), *i* = 1 … 8, the equation for the mass conservation is expressed as

(1) $$\frac{d}{dt}\int_{\Omega }s_{i}\left(x,t\right)dx=-\int_{\Sigma }\varphi_{i}\left(x,t\right)∙d\Sigma +\int_{\Omega }f_{i}\left(s\right)d\Omega \text{,}$$

and the divergence theorem gives

(2) $$\frac{\partial s_{i}}{\partial t}=-\nabla ∙\varphi_{i}+f_{i}\left(s\right)\text{.}$$

In the above eqs. (1), (2), ***φ***_*i*_ is the flux of *s*_*i*_ through the bounding surface *Σ*, *f*_*i*_ is the source term which depends on the species concentration. In particular eq. (2), is the mass balance equation expressed in Lagrangian form, represents the evolution equation for a generic species *s*_*i*_, and accounts for the mass conservation with the presence of a source term. Further, it is a parabolic PDE containing the diffusion of a species with concentration *s*_*i*_, and the flux ***φ***_*i*_ includes a diffusivity coefficient assumed as a constant. In general, the movement of biological species can be different from the motion of inorganic molecules, specifically diffusion and advection, because the interaction between the physics and the biological kinetics must be accounted for together with the influence on the motion of other biological agents. The Fick's law for the diffusive flux of a species through a surface is expressed in terms of a concentration gradient and of a specific diffusion coefficient. The advective motion depends on the velocity of the considered species inside a fluid, and for our purposes can be set to zero. For the dynamics of a given biological species, the interaction between biological kinetics and physics is characteristic of the problem under consideration and gives rise to the source/sink contributions in the reaction term. Moreover, the influence of other species on the motion of *s*_*i*_ gives rise to additional flux contributions among which, in the present work, we will consider chemotaxis terms. The latter are a consequence of chemical signaling originated by biological species producing a concentration gradient of emitted substances sensed by the diffusing species.

From here onwards with *p*_*S*_, *a*_*L*_, *a*_*N*_ and *a*_*IG*_, we refer to, respectively, the phagocytic score of M1, the IL-6 production rate, the IL-10 production rate and the IFN-γ production rate induced upon CAR-Ms administration, detected directly from the data reported in Ref. [38] as explained in the next Section.

The virions diffuse at a rate *D*_*V*_, are produced by *I* cells [11] at a rate *p* and eliminated at a rate *c* [42]. Moreover, are eliminated through phagocytosis by resident macrophages *M1* at a rate *ϕ*_11_, while the administered CAR-Ms induce an extra-phagocytosis contribution according to *p*_*s*_. The evolution equation for the virions is then given by:

(3) $$\frac{\partial V}{\partial t}=\nabla ∙\left(D_{V}\nabla V\right)+pI-cV-\phi_{11}M1-p_{s}\phi_{11}M1$$

It has been found that in critical COVID-19 patients a CD4^+^ T-cells depletion occurs [[43], [44], [45]], and it has been hypothesized that healthy CD4^+^ T-cells are attracted by infected CD4^+^ T-cells but are repelled by virions which can evade the immune machinery [12]. Then the taxis contribution to CD4^+^ T-cells dynamics includes, in addition to diffusion at a rate *D*_*T*_, contributions from opposite mechanisms [39,46], i.e., chemotaxis (attraction) and fugetaxis (repulsion), at *χ*_*I*_ and *χ*_*V*_ rates, respectively, where *T*_*M*_ denotes the maximum carrying capacity for healthy CD4^+^ T-cells. Other reaction terms include consumption by the interaction with virions, at a rate *k* [39,42], promotion by IL-6 [47,48] at a rate *ϕ*_21_, while the *a*_*L*_*ϕ*_21_*L* term accounts for the IL-6 extra contribution coming upon CAR-Ms administration. In the same way they are promoted by IFN-γ [[49], [50], [51]] at a rate *ϕ*_25_ and *a*_*IG*_*ϕ*_25_ but are inhibited by IL-10 [[51], [52], [53], [54]] at rates *ϕ*_23_ and *a*_*N*_*ϕ*_23_. CD4^+^ T-cells are promoted by M1 macrophages by means of direct [55] and indirect [56] mechanisms at a global rate *ϕ*_22_; instead, they are inhibited directly by M2 phenotypes [55], and indirectly because of the promotion of regulatory T cells (T_reg_), which have an inhibitory effect on CD4^+^ T-cells [57,58], at a global rate *ϕ*_24_. Hence, CD4^+^ T-cells evolve according to

(4) $$\frac{\partial T}{\partial t}=\nabla ∙\left\{D_{T}\nabla T-T\left[\chi_{I}\left(1-\frac{T}{T_{M}}\right)\nabla I-\chi_{V}\left(1-\frac{T}{T_{M}}\right)\nabla V\right]\right\}-kVT+\phi_{21}L+{a_{L}\phi }_{21}L+\phi_{22}M1-\phi_{23}N-{a_{N}\phi }_{23}N-\phi_{24}M2+\phi_{25}IG+{a_{IG}\phi }_{25}IG$$

Due to the lack of experimental measurements, we hypothesize that infected CD4^+^ T-cells diffuse at the same rate of healthy cells, so we put their diffusion coefficient *D*_*I*_ equal to *D*_*T*_. On the other hand, infected CD4^+^ T-cells are produced by SARS-CoV-2 virions infecting CD4^+^ T-cells at a rate *k*, while undergo depletion at a rate *δ* due to natural decay, and are inhibited by the interaction with the pro-inflammatory IL-6 molecules at a rate *ϕ*_*32*_ as a consequence of an increased pro-inflammatory environment [12,59]. In addition, a term accounting for the extra production of IL-6 due to CAR-Ms administration, *a*_*L*_*ϕ*_32_, is included,

(5) $$\frac{\partial I}{\partial t}=\nabla ∙\left(D_{I}\nabla I\right)+kVT-\delta I-\phi_{32}IL-a_{L}\phi_{32}IL\text{.}$$

The contribution to taxis of M1 macrophages has been hypothesized [12] as coming not only from random diffusion at a rate *D*_*M1*_, but also from chemotaxis because of infected CD4^+^ T-cells, at a rate *χ*_*I*_, with a maximum carrying capacity for M1 put to *M1*_*M*_. From a biological point of view, such choice is motivated by the clinical evidence that M1 infiltrate the infected tissue during the early stage of the COVID-19 disease [43], while a repolarization towards the M2 phenotype can occur later [2], even if it can be inhibited by the production of IL-6 and IFN-γ [60]. Moreover, M1 are produced by IL-6 and by IFN-γ [12,49,50], at rates *ϕ*_*41*_ and *ϕ*_*43*_, respectively, and inhibited during the inflammation resolution [6,51,52,61] by IL-10, at a rate *ϕ*_*42*_:

(6) $$\frac{\partial M1}{\partial t}=\nabla ∙\left[{D_{M1}\nabla M1-\chi }_{I}M1\left(1-\frac{M1}{{M1}_{M}}\right)\nabla I\right]+\phi_{41}L+{a_{L}\phi }_{41}L-\phi_{42}N-{a_{N}\phi }_{42}N+\phi_{43}IG+{a_{IG}\phi }_{43}IG\text{.}$$

The terms containing the parameters *a*_*L*_ and *a*_*IG*_, and *a*_*N*_, respectively, account for pro-inflammatory and anti-inflammatory contributions induced by CAR-Ms administration.

The spike protein of SARS-CoV-2 induces an inflammatory milieu through the hyper-production of IL-6 and IFN-γ [60,62], the latter promoting a repolarization of M2 towards M1 [63]. On the other hand, IL-10 induces the M1 polarization towards M2 [14,61], so the dynamical evolution of M2 macrophages includes the diffusion at a rate *D*_*M2*_, indirect promotion by IL-10 [51] at a rate *ϕ*_*51*_, deactivation by IL-6 and IFN-γ at rates [12], respectively, *ϕ*_*52*_ and *ϕ*_*53*_, to which is added the contribution deriving from the administration of CAR-Ms according to *a*_*N*_, *a*_*L*_, and *a*_*IG*_:

(7) $$\frac{\partial M2}{\partial t}=\nabla ∙\left(D_{M2}\nabla M2\right)+{\phi_{51}N+{a_{N}\phi }_{51}N-\phi }_{52}L-{a_{L}\phi }_{52}L-\phi_{53}IG-{a_{IG}\phi }_{53}IG\text{.}$$

The governing equation for IL-6 gives diffusion according to *D*_*L*_, production by M1 [2,5,13,55] at a rate *ϕ*_*61*_ and inhibition by IL-10 [13,51,52,61,64] at a rate *ϕ*_*63*_, and the contribution coming from CAR-Ms administration is accounted for by *a*_*N*_:

(8) $$\frac{\partial L}{\partial t}=\nabla ∙\left(D_{L}\nabla L\right)+\phi_{61}M1-\phi_{63}N-{a_{N}\phi }_{63}N\text{.}$$

Similarly, the model equation for IL-10 includes diffusion at rate *D*_*N*_, promotion by M2 [2,52] at a rate *ϕ*_*71*_ and inhibition by pro-inflammatory IL-6 [13] according to *ϕ*_*73*_, while the extra contribution from CAR-Ms is accounted for by *a*_*L*_:

(9) $$\frac{\partial N}{\partial t}=\nabla ∙\left(D_{N}\nabla N\right)+\phi_{71}M2-\phi_{73}L-{a_{L}\phi }_{73}L\text{.}$$

The last model equation concerns the evolution of IFN-γ, contribution to which includes diffusion according to *D*_*IG*_, production/activation by CD4^+^ T-cells [49,50,55] and M1 macrophages [1,4] at rates *ϕ*_*81*_ and *ϕ*_*82*_, respectively, inhibition by IL-10 [50] at a rate *ϕ*_*83*_, and the extra contribution from CAR-Ms administration according to *a*_*N*_:

(10) $$\frac{\partial IG}{\partial t}=\nabla ∙\left(D_{IG}\nabla IG\right)+\phi_{81}T+\phi_{82}M1-\phi_{83}N-{a_{N}\phi }_{83}N\text{.}$$

## 3 Materials and methods

We consider a portion of biological tissue constituted by a square of human respiratory tract (HRT) with side 0.1 cm, coinciding with the physical domain *Ω* in which we monitor the dynamical evolution of the PDE system eqs. (3), (4), (5), (6), (7), (8), (9), (10). Zero-flux boundary conditions are imposed assuming that all the variables are confined within the biological domain. Typical estimates [12,65,66] of the reference values are summarized in Table 1, evidencing that the sum of administrated CAR-Ms and pre-existing M1 macrophages is *M1*_*r*_ = 6.9 × 10^6^ n cm^−3^. The *T*_*M*_ and *M1*_*M*_ values in eqs. (4), (6) have been set equal to the reference quantities *T*_*r*_ = 5 × 10^6^ n cm^−3^ and *M1*_*r*_, respectively.

**Table 1:** Summary of the reference quantities used for the model non-dimensionalization.

Reference Quantity	Symbol	Units	Value
Virus (*V*)	*V*_*r*_	n cm^−3^	1 × 10^7^
T cells	*T*_*r*_	cell cm^−3^	5 × 10^6^
T maximum carrying capacity	*T*_*M*_	cell cm^−3^	5 × 10^6^
I cells	*I*_*r*_	cell cm^−3^	5 × 10^5^
M1 macrophages	*M1*_*r*_	cell cm^−3^	6.9 × 10^6^
M1 maximum carrying capacity	*M1*_*M*_	cell cm^−3^	6.9 × 10^6^
M2 macrophages	*M2*_*r*_	cell cm^−3^	6.9 × 10^6^
IL-6 (*L*)	*L*_*r*_	n cm^−3^	2.87 × 10^9^
IL-10 (*N*)	*N*_*r*_	n cm^−3^	2.87 × 10^9^
IFN-γ (*IG*)	*IG*_*r*_	n cm^−3^	3.58 × 10^9^
Characteristic Length	*l*	cm	0.1
Characteristic Diffusion Coefficient	*D*	cm^2^ s^−1^	1 × 10^−6^
Characteristic Time Scale	*τ*	s	1 × 10^4^

The parameters introduced in the model are summarized in Table 2 in non-dimensional form, while their estimation and the procedure for their non-dimensionalization are detailed in the Supplementary Material.

**Table 2:** Summary of the parameters used in the model.

Description	Symbol	Units	Non-Dimensional Parameter	Value
*V* diffusion coefficient	*D*_*V*_	cm^2^ s^−1^	*D*_*V*_*D*^−1^	1 × 10^−2^
*T* diffusion coefficient	*D*_*T*_	cm^2^ s^−1^	*D*_*T*_*D*^−1^	5 × 10^−3^
*I* diffusion coefficient	*D*_*I*_	cm^2^ s^−1^	*D*_*I*_*D*^−1^	5 × 10^−3^
*M1* diffusion coefficient	*D*_*M1*_	cm^2^ s^−1^	*D*_*M1*_*D*^−1^	5 × 10^−5^
*M2* diffusion coefficient	*D*_*M2*_	cm^2^ s^−1^	*D*_*M2*_*D*^−1^	5 × 10^−5^
*L* diffusion coefficient	*D*_*L*_	cm^2^ s^−1^	*D*_*L*_*D*^−1^	1.45 × 10^−2^
*N* diffusion coefficient	*D*_*N*_	cm^2^ s^−1^	*D*_*N*_*D*^−1^	1.45 × 10^−2^
IG diffusion coefficient	*D*_*IG*_	cm^2^ s^−1^	*D*_*IG*_*D*^−1^	1.45 × 10^−2^
*V* production coefficient	*p*	s^−1^	*pτI*_*r*_*V*_*r*_^*−*^*^1^*	1.16 × 10^−1^
*V* clearing coefficient	*c*	s^−1^	*cτ*	6.94 × 10^−2^
*M1* phagocytosis coefficient	*ϕ*_*11*_	s^−1^	*ϕ*_*11*_*τM1*_*r*_*V*_*r*_^*−*^*^1^*	1 × 10^−3^
*I* chemotactic coefficient	*χ*_*I*_	cm^5^ s^−1^ cell^−1^	*χ*_*I*_*I*_*r*_*D*^*−*^*^1^*	1 × 10^−3^
*V* fugetactic coefficient	*χ*_*V*_	cm^5^ s^−1^ cell^−1^	*χ*_*V*_*V*_*r*_*D*^*−*^*^1^*	5 × 10^−2^
*T* infection rate	*k*	cm^3^ s^−1^ cell^−1^	*kτV*_*r*_	7.4 × 10^−4^
*T* activation rate by L	*ϕ*_*21*_	s^−1^	*ϕ*_*21*_*τL*_*r*_*T*_*r*_^*−*^*^1^*	11.5
*T* production rate by M1	*ϕ*_*22*_	s^−1^	*ϕ*_*22*_*τM1*_*r*_*T*_*r*_^*−*^*^1^*	2.3 × 10^7^
*T* inhibition rate by N	*ϕ*_*23*_	s^−1^	*ϕ*_*23*_*τN*_*r*_*T*_*r*_^*−*^*^1^*	22.96
*T* inhibition rate by M2	*ϕ*_*24*_	s^−1^	*ϕ*_*24*_*τM2*_*r*_*T*_*r*_^*−*^*^1^*	0.95 × 10^5^
*T* production rate by IG	*ϕ*_*25*_	s^−1^	*ϕ*_*25*_*τIG*_*r*_*T*_*r*_^*−*^*^1^*	1 × 10^−3^
*I* decay rate	*δ*	s^−1^	*δτ*	2 × 10^−2^
*I* reduction rate by hyper-inflammation	*ϕ*_*32*_	cm^3^ s^−1^ cell^−1^	*ϕ*_*32*_*τL*_*r*_	10
*M1* production rate by L	*ϕ*_*41*_	s^−1^	*ϕ*_*41*_*τL*_*r*_*M1*_*r*_^*−*^*^1^*	1 × 10^−3^
*M1* inhibition rate by N	*ϕ*_*42*_	s^−1^	*ϕ*_*42*_*τN*_*r*_*M1*_*r*_^*−*^*^1^*	4 × 10^−4^
*M1* production rate by IG	*ϕ*_*43*_	s^−1^	*ϕ*_*43*_*τIG*_*r*_*M1*_*r*_^*−*^*^1^*	1 × 10^−11^
*M2* promotion rate by N	*ϕ*_*51*_	s^−1^	*ϕ*_*51*_*τN*_*r*_*M2*_*r*_^*−*^*^1^*	2.3 × 10^−2^
*M2* inhibition rate by L	*ϕ*_*52*_	s^−1^	*ϕ*_*52*_*τL*_*r*_*M2*_*r*_^*−*^*^1^*	6.5 × 10^−3^
*M2* inhibition rate by IG	*ϕ*_*53*_	s^−1^	*ϕ*_*53*_*τIG*_*r*_*M2*_*r*_^*−*^*^1^*	1 × 10^−11^
*L* production rate by M1	*ϕ*_*61*_	s^−1^	*ϕ*_*61*_*τM1*_*r*_*L*_*r*_^*−*^*^1^*	5 × 10^−1^
*L* inhibition rate by N	*ϕ*_*63*_	s^−1^	*ϕ*_*63*_*τN*_*r*_*L*_*r*_^*−*^*^1^*	2 × 10^−3^
*N* production rate by M2	*ϕ*_*71*_	s^−1^	*ϕ*_*71*_*τM2*_*r*_*N*_*r*_^*−*^*^1^*	1 × 10^−1^
*N* inhibition rate by L	*ϕ*_*73*_	s^−1^	*ϕ*_*73*_*τL*_*r*_*N*_*r*_^*−*^*^1^*	9.26 × 10^−5^
*IG* production rate by T	*ϕ*_*81*_	s^−1^	*ϕ*_*81*_*τT*_*r*_*IG*_*r*_^*−*^*^1^*	2 × 10^−1^
*IG* production rate by M1	*ϕ*_*82*_	s^−1^	*ϕ*_*82*_*τM1*_*r*_*IG*_*r*_^*−*^*^1^*	5 × 10^−2^
*IG* inhibition rate by N	*ϕ*_*83*_	s^−1^	*ϕ*_*83*_*τN*_*r*_*IG*_*r*_^*−*^*^1^*	1 × 10^−3^

At *t* = *t*_*0*_ the system is administered with the CAR-Ms, assuming that the SARS-CoV-2 virions already infect the domain at ***x*** = (0,0) with a gaussian distribution having standard deviation σ = 0.1, hence *V*(***x***,0) = exp(-0.5|***x***|^2^*σ*^−2^); the infected CD4^+^ T-cells represent a fraction of initial virions according to *I*(***x***,0) = 0.5*V*(***x***,0), hence the initial distribution of healthy CD4^+^ T-cells is *T*(***x***,0) = 1 - 0.5*V*(***x***,0). Both M1 and M2 macrophages are initially distributed around the origin with gaussian shape with *σ* = 0.1, hence *M1*(***x***,0) = 0.05exp(-0.5|***x***|^2^*σ*^−2^) and *M2*(***x***,0) = 0.1exp(-0.5|***x***|^2^*σ*^−2^). The initial distributions of IL-6 and IFN-γ follow that of M1, then *L*(***x***,0) = 0.05 exp(-0.5|***x***|^2^*σ*^−2^) and *IG*(***x***,0) = 0.05 exp(-0.5|***x***|^2^*σ*^−2^), finally *N*(***x***,0) = 0.1exp(-0.5|***x***|^2^*σ*^−2^) since we assume that IL-10 initial distribution follows that of M2. The model simulation has been carried out on a non-dimensional domain *Ω* = [0,1] × [0,1] which refers to a physical square with side *l* = 0.1 cm where typical diffusivities are in the order of *D* = 1 × 10^−6^ cm^2^ s^−1^, from which a reference value *τ* = *l*^2^*D*^−1^ = 1 × 10^4^ s is obtained for the non-dimensionalization of the time scale. Computations were performed for *t* ∈ [0,60] with step δ*t* = 0.1, while the dimensional time values are recovered by multiplying the non-dimensional values by *τ*.

Particular attention deserves the procedure used to estimate the boosting of the phagocytic activity and of ILs production induced by CAR-Ms. For this purpose, we used the experimental results reported in Fu et al. [38], Fig. 2, detecting, for CAR_γ_, CAR_MGF10_, CAR_MERTK_ and CAR_ζ_, the intensity of the phagocytic score and of the quantities of the IL-6, IL-10 and IFN-γ produced, normalizing each value to that corresponding for control untransduced (UTD) macrophages reported in the same Fig. 2. We are confident that possible errors due to the used procedure fall within the reported experimental uncertainty. The values so obtained are the coefficients introduced in the model equations named *p*_*S*_, *a*_*IG*,_ *a*_*L*_, and *a*_*N*_, identified as the CAR parameters and reported in Table 3, referring to, respectively, phagocytic score, IFN-γ overproduction, IL-6 overproduction, and IL-10 overproduction.

**Table 3:** **Summary of the boosting parameters.** The details for the estimation procedure are described in the text.

	Boosting parameters			
	Phagocytic score (*p*_*S*_)	IFN-γ (*a*_*IG*_)	IL-6 (*a*_*L*_)	IL-10 (*a*_*N*_)
CAR_γ_	82	4	3.25	5
CAR_MGF10_	76	2.5	2	2.5
CAR_MERTK_	97	0.5	0.75	1.5
CAR_ζ_	93	4	3.125	4.25

To monitor the dynamical evolution of the system we solved the PDEs system using the finite element method (FEM) [67] as implemented via the COMSOL Multiphysics ™ package: the PDEs have been uniformly discretized on the square domain with a mesh constituted by 17956 square elements, while the variable distribution inside each element has been interpolated with quadratic shape functions. For the time discretization a backward differentiation method has been used, as it ensures unconditional stability [65] in highly non-linear systems [68], resulting in a robust computation as changes of the parameter values within ±10 % did not produced appreciable changes to the variables profiles at *t* = 60. Some details on the FEM implementation of the solution procedure can be found in the Supplementary Material. Given the structure of each model equation, at the lower time steps the reaction terms accounting for contributions coming from CAR-Ms could act like sink terms: for such reason, to prevent some concentration to be pushed towards negative values devoid of any physical and biological meaning, we impose that each concentration can assume only non-negative values.

## 4 Results and discussion

**Fig. 1:**
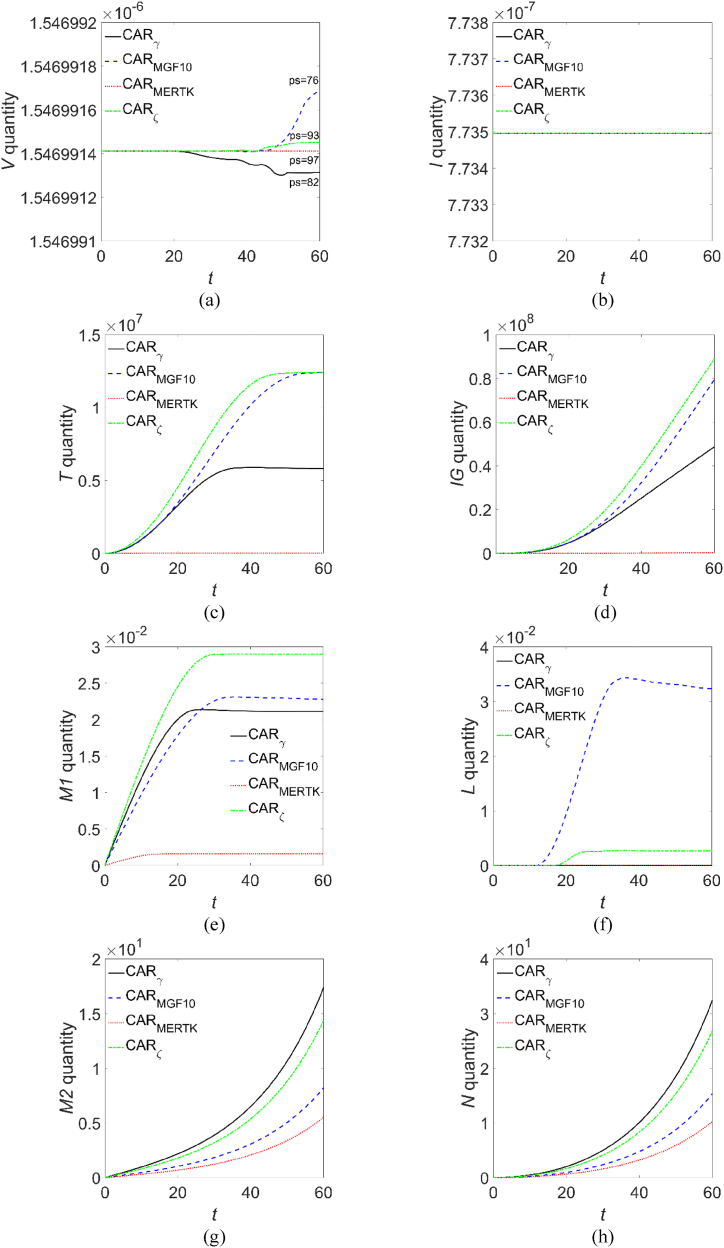
**Evolution of the species present in the domain.** In each panel, the solid black line refers to CAR_γ_, the dotted red line refers to CAR_MERTK_, the dash-dotted green line refers to CAR_ζ_, and dashed blue line refers to CAR_MGF10_: (a) virions, (b) infected CD4^+^ T cells, (c) healthy CD4^+^ T cells, (d) IFN-γ molecules, (e) M1 macrophages, (f) IL-6 molecules, (g) M2 macrophages, (h) IL-10 molecules. (For interpretation of the references to colour in this figure legend, the reader is referred to the Web version of this article.)

For each considered CAR-M, the concentrations dynamics of the species were computed and then numerically integrated in space to obtain the temporal evolution shown in Fig. 1. For *t* ∈ [0,20] the four CAR constructs exhibit similar clearance activity against the virions, according to the experimental results reported in Fu et al. [38]. For *t* > 20, although in a narrow range, the quantity of virions evolves differently depending on the CAR-M administered, and the best clearance activity is achieved with CAR_γ_, even if they have a lower phagocytic score (82) with respect to CAR_MERTK_ (97), but also with respect to CAR_ζ_ (93). The evolution of infected CD4^+^ T-cells remains constant over time within the graphical resolution, thus indicating a comparable effectiveness of the four CAR-Ms immunotherapies in containing the spread of infection. The healthy CD4^+^ T-cells evolve towards saturation values, except for CAR_MERTK_ administration for which no production is shown, while a limited production is achieved after CAR_γ_ administration. Similarly, no detectable IFN-γ production can be observed with CAR_MERTK_ administration, while with the other CARs the production increases, almost linearly for *t* > 25, progressively steeper on administering the system with CAR_γ_, CAR_MGF10_ and CAR_ζ_. The production of the M1 macrophages is minimal with CAR_MERTK_ administration, growing for the other CAR-Ms, but in all cases the total amount of M1 cells evolves towards saturation values. Very interesting appears the evolution of the IL-6 quantity for reasons that will be discussed later, for which the lowest production is achieved with both the administration of CAR_MERTK_ and CAR_γ_. The M2 production is the lowest upon CAR_MERTK_ administration and is the highest with CAR_γ_, a trend reflecting also in the IL-10 evolution.

**Fig. 2:**
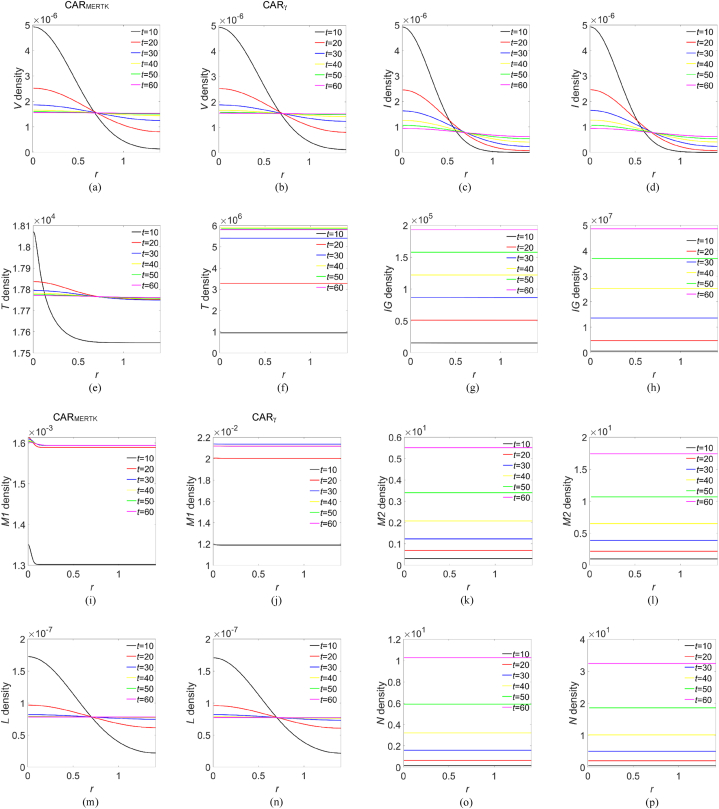
**Computed cross-sectional densities of the species present in the domain.** Profiles were obtained along the diagonal cutline (see text). The curves in each panel were extracted for *t* ∈ [10,20,30,40,50,60]. First and second column refer to CAR_MERTK_ and to CAR_γ_, respectively: (a)–(b) virions, (c)–(d) infected CD4^+^ T cells, (e)–(f) healthy CD4^+^ T cells, (g)–(h) IFN-γ molecules, (i)–(j) M1 macrophages, (k)–(l) M2 macrophages, (m)–(n) IL-6 molecules, (o)–(p) IL-10 molecules.

The results reported in Ref. [38] come from *in vitro* experiments and refer to the response of cell lines few hours after inoculation with a SARS-CoV-2 pseudotyped virus, from which the Authors deduce that CAR_MERTK_ immunotherapy induces the best SARS-CoV-2 clearance with the lowest ILs production, and therefore identify these engineered CARs as the best choice for the treatment of COVID-19 patients since high ILs values are associated with poor prognosis [35,36]. Our results, instead, refer to the dynamical evolution of the SARS-CoV-2 virions during their interaction with the immune system, starting from the administration of the CAR-Ms up to almost a week later. As can be seen from the virions evolution, the simulations predict that a good clearance capacity is obtained with CAR_MERTK_ (*p*_*S*_ = 97), but CAR_γ_ (*p*_*S*_ = 82) performs even better, from which we deduce that during the interaction of SARS-CoV-2 with the immune system, not only the phagocytic performance but also the induced cytokines production may play a primary role against the COVID-19 disease. Hence, in the following, we will restrict our attention to the dynamical evolution of CAR_MERTK_ and CAR_γ_ immunotherapies, as they exhibit the more interesting characteristics regarding the virus clearance capacity.

In Supplementary Figs. S1–S8 we show the computed densities for each species, simulating the administration of CAR_MERTK_ (first column) and CAR_γ_ (second column). The 2D density maps are plotted in colour scale and a 3D rendering for better readability. In all cases, at *t* = 0 the density distribution is imposed by the initial conditions while for visualization purposes we extracted the data at *t* = 10 (∼1.16 days), *t* = 20 (∼2.31 days), *t* = 30 (∼3.47 days), *t* = 40 (∼4.63 days), *t* = 50 (∼5.79 days) and *t* = 60 (∼6.94 days). Starting from *t* = 10, and up to *t* = 60, the virions density decreases at the origin while infiltrating the domain, Supplementary Fig. S1, with a behaviour qualitatively similar for both CAR-Ms, while the maps in Supplementary Fig. S2 refer to the infected CD4^+^ T-cells that exhibit a trend consequent to the virus progression. The density of healthy CD4^+^ T-cells, instead, evolves differently depending on the administered CAR-M: in fact, in Supplementary Fig. S3 a low production upon CAR_MERTK_ administration is observed, while the effect of CAR_γ_ is capable to induce a growing CD4^+^ T-cells production which infiltrate the domain uniformly. A similar evolution is observed for M1 macrophages in Supplementary Fig. S4, while, for the M2 phenotype a modest infiltrative dynamics is observed upon CAR_MERTK_ administration, see Supplementary Fig. S5. The evolution of IL-6 density, Supplementary Fig. S6, does not show significant differences as a function of the administered CAR-M, in both intensity and domain infiltration, highlighting what has been already observed in the quantity evolution shown in Fig. 1. The dynamical evolution of IL-10 and IFN-γ, Supplementary Figs. S7 and S8, respectively, shows a progressive intensity growth and domain infiltration upon CAR_γ_ administration; for CAR_MERTK_ administration, instead, the IL-10 grows and infiltrates weakly, while the growth of IFN-γ remains close to the origin. Alongside this qualitative information, further details can be obtained via the analysis of the density profiles extracted along a preferred direction of the simulated domain: due to the domain geometry, we choose the diagonal cut line from ***x*** = (0,0) to ***x*** = (1,1). The density profiles shown in Fig. 2, referring to CAR_MERTK_ and CAR_γ_ administration, first and third, and second and fourth columns, respectively, put in evidence as both immunotherapies are capable to contain the virions spread across the domain, even if a slight domain infiltration is observed. Similarly, the spread of infected CD4^+^ T-cells is contained and posed to decrease across the domain, signal of a possible virulence dampening, in agreement with the quantity evolution in Fig. 1. Quite different, instead, appears the healthy CD4^+^ T-cells evolution between the two immunotherapies: with CAR_MERTK_ administration their density decreases at the origin while infiltrating the domain, with CAR_γ_ administration, instead, they are uniformly distributed and growing across the domain. The IFN-γ dynamics for both CAR-Ms administration exhibits a quite uniform growth and infiltration, like, moreover, that of M1, M2 and IL-10. Concerning the IL-6 profiles, we are in presence of overlapping distributions for both immunotherapies: at *t* = 10 the molecules are clustered in a broad structure having its maximum at the origin, suddenly decreasing for *t* ≥ 20 while infiltrating the domain. Such behaviour is consistent with the evolution of the species shown in Fig. 1, suggesting that the highest virions clearance is associated to the CAR immunotherapies inducing the lower IL-6 production, i.e., CAR_MERTK_ and CAR_γ_. It should be noted, however, that the best virions clearance is obtained with CAR_γ_ administration and is associated to the highest production of anti-inflammatory agents such as M2 and IL-10, and above all with a higher production of CD4^+^ T-cells and IFN-γ with respect to the CAR_MERTK_ administration.

**Fig. 3:**
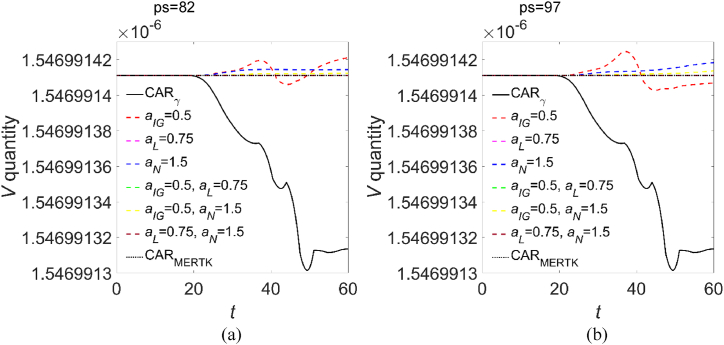
**Evolution of the SARS-CoV-2 virions with virtualized parameters.** In each panel, solid and dotted black lines refer to the virions evolution upon CAR_γ_ and CAR_MERTK_ administration, respectively, as in Fig. 1 but reported here for reference. The other curves have been obtained varying the CAR_γ_ parameters, individually and in combination with each other, towards CAR_MERTK_. The evolution of the virions quantity has been obtained imposing a phagocytic score of (a) ps = 82, and (b) ps = 97.

With the aim of identifying the master regulator of the virions clearance, we performed a virtualization of the two immunotherapies: starting from the engineered CAR_γ_, the CAR parameters of Table 3 were varied, both individually and in combination with each other, making CAR_γ_ virtually tend to CAR_MERTK_, assuming that each virtualized immunotherapy be administered at *t* = 0. In Fig. 3 we show the resulting virions evolution, where left and right panels refer to virtualization with, respectively, *p*_*S*_ = 82 and *p*_*S*_ = 97. In each panel, the curves referring to the quantities obtained for non-virtualized CAR_γ_ and CAR_MERTK_ macrophages are reported for reference. As it can be seen from both panels, is the concurrent work of all cytokines that determines the better clearance function, regardless of the phagocytic score, as lowering the CAR_γ_ parameters towards CAR_MERTK_ values, even for a single cytokine, the clearance capacity of the virtual CAR worsen, approaching that of CAR_MERTK_. On the other hand, the experimental work of Patra and Ray on COVID-19 patients [70], where a synergistic effect between phagocytic capacity and cytokines production has been found, gives further support to our findings.

In a time lapse up to about 2.8 days from CAR-Ms administration no differences are predicted by the model in the virions clearance capacity, while all the considered immunotherapies should be able to contain the virus proliferation and the infection of CD4^+^ T-cells, see Fig. 1. For *t* > 2.8 days, instead, the best clearance activity pertains to CAR_γ_ macrophages, which induce an IL-6 production as low as CAR_MERTK_ but a higher production of healthy CD4^+^ T-cells, IL-10 and IFN-γ. The latter is recognized for its macrophage-promoting and antiviral activity [49,50,69] and for being a phagocytosis enhancer [71], while IL-10, given its pleiotropic nature [9,10], in the present context may act as an anti-inflammatory IL. The profiles of Fig. 2 tell us that the densities of virions and infected CD4^+^ T-cells evolve in a similar way, that is towards a contained infection, and are strongly correlated to the density profiles of IL-6, for both immunotherapies. The spatial distribution of other immune components shows an infiltrative dynamics, more pronounced with the CAR_γ_ immunotherapy. It should be noted that, except for IL-6, the pro-inflammatory response about one week after CAR_γ_ administration, i.e., CD4^+^ T-cells, IFN-γ and M1 macrophages, is one or two order of magnitude greater than after CAR_MERTK_ administration. In particular, from the curves shown in Fig. 2, but also from those of Fig. 1, we can deduce that, at *t* = 60, administering CAR_MERTK_ the production of CD4^+^ T-cells, IFN-γ and IL-10 is reduced by, respectively, 99.7 %, 99.6 % and 69 %, compared to the administration of CAR_γ_. The anti-inflammatory response represented by M2 and IL-10 density profiles, instead, is always comparable for both immunotherapies. Therefore, considering the virtualization results above discussed, we can conclude that CAR_γ_ immunotherapy trigger the immune machinery orchestrating the best clearing activity of the SARS-CoV-2 virions.

## 5 Conclusion

The proposed model reproduces the experimental results of Fu et al. [38], as confirmed by our computational results that advance the understanding of the mechanism governing the interaction of CAR constructs with SARS-CoV-2 virions, whose efficacy in fighting the COVID-19 disease is based on the cooperation of cytokines, not only on the phagocytic capacity of macrophages, even if the latter, in the case of CAR_γ_ administration, can be enhanced by the production of cytokines. If CAR_MERTK_ macrophages provide a good clearance of virions with a low production of ILs, in the long run CAR_γ_ show a better capacity to contain virions with an equally low production of pro-inflammatory IL-6, and with a higher production, besides of CD4^+^ T-cells, of IFN-γ and IL-10 which induce, respectively, antiviral and healing effects. Therefore, the above results suggest CAR_γ_ immunotherapy as suitable for the treatment of COVID-19 disease and therefore worth of further clinical investigation. Future work will be directed to the extension of the model by including other contributions to the SARS-CoV-2 dynamics during its interaction with the immune system, such as those coming from tumour necrosis factor-alpha, a well-known master regulator cytokine triggering the pro-inflammatory response of the immune system, having implications also for the oncologic disease.

## Data and code availability statement

Data will be made available on request.

## Declaration of Competing Interest

The authors declare that they have no known competing financial interests or personal relationships that could have appeared to influence the work reported in this paper.
