# Supplementary material for: In silico assessment of CAR macrophages activity against SARS-CoV-2 infection
Source: Heliyon. 2024 Oct 23;10(21):e39689. doi: 10.1016/j.heliyon.2024.e39689 (PMC11550025; doi:10.1016/j.heliyon.2024.e39689)
Supplement: Multimedia component 1 [file mmc1.docx]

SUPPLEMENTARY MATERIAL

***In silico* assessment of CAR Macrophages activity against SARS-CoV-2 infection.**

Antonino Amoddeo

Department of Civil, Energy, Environment and Materials Engineering, Università ’Mediterranea’ di Reggio Calabria, Via R. Zehender 1, Feo di Vito, I-89122 Reggio Calabria, Italy

**Correspondence:** antonino.amoddeo@unirc.it; Tel: +39-0965-169-3299; Fax: +39-0965-169-2201.

**1. Reference quantities and Parameters**

Several parameters and reference quantities have been taken from ref. [1], here we detail the quantities introduced *ex novo* as coming from the modelling of the IFN-γ dynamics and of the overproduction characterizing the CAR-Ms or modified from [1]. We refer to the ‘calibrated’ parameter as the one which value has been refined, within a range of coherent values, to reproduce the experimental findings we are modelling.

a) References quantities

To non-dimensionalize the PDE system we introduce a set of reference values for the biological quantities [2], and for space and time, summarized in the Supplementary Table S1. In particular, space and time have been non-dimensionalized by dividing, respectively, a distance by *l* = 0.1 cm, and a time by *τ* = 1 × 10^4^ s, recognizing that the reference diffusion coefficient *D* = *l*^2^*τ*^−1^. We assume that the virions can infect a fraction of CD4^+^ - T cells, hence *I_r_* = 5 × 10^5^ cell cm^-3^.

| **Reference Quantity** | **Symbol** | **Units** | **Value** | **Source** |
| --- | --- | --- | --- | --- |
| Virus (*V*) | *V_r_* | N cm^-3^ | 1 × 10^7^ | [1] |
| T cells | *T_r_* | cell cm^-3^ | 5 × 10^6^ | [1] |
| T maximum carrying capacity | *T_M_* | cell cm^-3^ | 5 × 10^6^ | [1] |
| I cells | *I_r_* | cell cm^-3^ | 5 × 10^5^ | assumed |
| M1 macrophages | *M1_r_* | cell cm^-3^ | 6.9 × 10^6^ | [1] |
| M1 maximum carrying capacity | *M1_M_* | cell cm^-3^ | 6.9 × 10^6^ | [1] |
| M2 macrophages | *M2_r_* | cell cm^-3^ | 6.9 × 10^6^ | [1] |
| IL-6 (*L*) | *L_r_* | n cm^-3^ | 2.87 × 10^9^ | [1] |
| IL-10 (*N*) | *N_r_* | n cm^-3^ | 2.87 × 10^9^ | [1] |
| IFN-γ (*IG*) | *IG_r_* | n cm^-3^ | 3.58 × 10^9^ | [3], [4] |
| Characteristic Length | *l* | cm | 0.1 | [1] |
| Characteristic Diffusion Coefficient | *D* | cm^2^ s^-1^ | 1 × 10^-6^ | [1] |
| Characteristic Time Scale | *τ* | s | 1 × 10^4^ | [1] |

**Supplementary Table S1. Summary of the reference quantities used for the model non-dimensionalization.**

We refer to the paper of Liao *et al.* [3] on the mathematical modelling of the anti-tumour response induced by interleukin-27 (IL-27), involving also CD8^+^ -T cells, IL-10 and IFN-γ, for the reference quantity of, among others, IFN-γ, for which it is assumed the value of 10^2^ pg cm^-3^: since the molecular mass of IFN-γ, M_IFN-γ_ ≈ 16.8 kDa [4], the Avogadro number N_A_ = 6.022×10^23^ n mol^-1^, then the number of IFN-γ molecules contained in 10^2^ pg is given by

$\frac{mass (g)}{molecular mass (Da)}\times N_{A}=\frac{{10}^{-10}}{16.8\times{10}^{3}}\frac{g}{Da}\times6.022\times{10}^{23}\left( n {mol}^{-1} \right)\approx3.58\times{10}^{9} n$.

Therefore, we assume *IG_r_* = $3.58\times{10}^{9}$ n cm^-3^.

b) Parameters

Supplementary Table S2 summarizes the parameters introduced in the model, in which we insert: a short description of each parameter, the pertaining symbol, the pertaining units, the non-dimensional form, the non-dimensional value and finally the reference source, or a reference to the evaluation method. Regarding the non-dimensional parameter values, they are obtained by multiplying the dimensional values by the appropriate reference values already listed in Supplementary Table S1. So, for example, to recover the dimensional value of the virus diffusion coefficient in the first row we proceed as:

*D_V_D*^-1^ = 1×10^-2^, then *D_V_* = 1×10^-2^×1×10^-6^ cm s^-1^ = 1 × 10^-8^ cm s^-1^.

According to Liao *et al.* [3], the value of the diffusion coefficient for IFN-γ is assumed as 1.25 × 10^-3^ cm^2^ day^-1^, then *D_IG_* = 1.45 × 10^-8^ cm^2^ s^-1^.

In Marée *et al.* [5] the phagocytosis rates of macrophages from normal and diabetes-prone mice are evaluated: considering the coefficients calculated for engulfment, and the procedure described for the unit conversion, we observe that the dimensional coefficient values for engulfment fall in the 10^-6^ s^-1^ range. We consider these values high, and in compliance with a precautionary principle, in view of the extra contribution due to CAR-Ms, we assume the value of the phagocytosis coefficient *φ_11_* = 1.45 × 10^-7^ s^-1^.

| **Description** | **Symbol** | **Units** | **Non-Dimensional**  **Parameter** | **Value** | **Source** |
| --- | --- | --- | --- | --- | --- |
| *V* diffusion coefficient | *D_V_* | cm^2^ s^-1^ | *D_V_D*^-1^ | 1×10^-2^ | [1] |
| *T* diffusion coefficient | *D_T_* | cm^2^ s^-1^ | *D_T_D*^-1^ | 5×10^-3^ | [1] |
| *I* diffusion coefficient | *D_I_* | cm^2^ s^-1^ | *D_I_D*^-1^ | 5×10^-3^ | [1] |
| *M1* diffusion coefficient | *D_M1_* | cm^2^ s^-1^ | *D_M1_D*^-1^ | 5×10^-5^ | [1] |
| *M2* diffusion coefficient | *D_M2_* | cm^2^ s^-1^ | *D_M2_D*^-1^ | 5×10^-5^ | [1] |
| *L* diffusion coefficient | *D_L_* | cm^2^ s^-1^ | *D_L_D*^-1^ | 1.45×10^-2^ | [1] |
| *N* diffusion coefficient | *D_N_* | cm^2^ s^-1^ | *D_N_D*^-1^ | 1.45×10^-2^ | [1] |
| IG diffusion coefficient | *D_IG_* | cm^2^ s^-1^ | *D_IG_D*^-1^ | 1.45×10^-2^ | [3] |
| *V* production coefficient | *p* | s^-1^ | *pτI_r_V_r_^-1^* | 1.16×10^-1^ | [1] |
| *V* clearing coefficient | *c* | s^-1^ | *cτ* | 6.94×10^-2^ | [1] |
| *M1* phagocytosis coefficient | *ϕ_11_* | s^-1^ | *ϕ_11_τM1_r_V_r_^-1^* | 1×10^-3^ | [5], assumed |
| *I* chemotactic coefficient | *χ_I_* | cm^5^ s^-1^ cell^-1^ | *χ_I_I_r_D^-1^* | 1×10^-3^ | [1] |
| *V* fugetactic coefficient | *χ_V_* | cm^5^ s^-1^ cell^-1^ | *χ_V_V_r_D^-1^* | 5×10^-2^ | [1] |
| *T* infection rate | *k* | cm^3^ s^-1^ cell^-1^ | *kτV_r_* | 7.4×10^-4^ | [1] |
| *T* activation rate by L | *ϕ_21_* | s^-1^ | *ϕ_21_τL_r_T_r_^-1^* | 11.5 | [1] |
| *T* production rate by M1 | *ϕ_22_* | s^-1^ | *ϕ_22_τM1_r_T_r_^-1^* | 2.3×10^7^ | [1] |
| *T* inhibition rate by N | *ϕ_23_* | s^-1^ | *ϕ_23_τN_r_T_r_^-1^* | 22.96 | [1] |
| *T* inhibition rate by M2 | *ϕ_24_* | s^-1^ | *ϕ_24_τM2_r_T_r_^-1^* | 0.95×10^5^ | [1] |
| *T* production rate by IG | *ϕ_25_* | s^-1^ | *ϕ_25_τIG_r_T_r_^-1^* | 1×10^-3^ | calibrated |
| *I* decay rate | *δ* | s^-1^ | *δτ* | 2×10^-2^ | [1] |
| *I* reduction rate by hyper-inflammation | *ϕ_32_* | cm^3^ s^-1^ cell^-1^ | *ϕ_32_τL_r_* | 10 | [1] |
| *M1* production rate by L | *ϕ_41_* | s^-1^ | *ϕ_41_τL_r_M1_r_^-1^* | 1×10^-3^ | [1] |
| *M1* inhibition rate by N | *ϕ_42_* | s^-1^ | *ϕ_42_τN_r_M1_r_^-1^* | 4×10^-4^ | calibrated |
| *M1* production rate by IG | *ϕ_43_* | s^-1^ | *ϕ_43_τIG_r_M1_r_^-1^* | 1×10^-11^ | calibrated |
| *M2* promotion rate by N | *ϕ_51_* | s^-1^ | *ϕ_51_τN_r_M2_r_^-1^* | 2.3×10^-2^ | calibrated |
| *M2* inhibition rate by L | *ϕ_52_* | s^-1^ | *ϕ_52_τL_r_M2_r_^-1^* | 6.5×10^-3^ | calibrated |
| *M2* inhibition rate by IG | *ϕ_53_* | s^-1^ | *ϕ_53_τIG_r_M2_r_^-1^* | 1×10^-11^ | calibrated |
| *L* production rate by M1 | *ϕ_61_* | s^-1^ | *ϕ_61_τM1_r_L_r_^-1^* | 5×10^-1^ | [1] |
| *L* inhibition rate by N | *ϕ_63_* | s^-1^ | *ϕ_63_τN_r_L_r_^-1^* | 2×10^-3^ | calibrated |
| *N* production rate by M2 | *ϕ_71_* | s^-1^ | *ϕ_71_τM2_r_N_r_^-1^* | 1×10^-1^ | [1] |
| *N* inhibition rate by L | *ϕ_73_* | s^-1^ | *ϕ_73_τL_r_N_r_^-1^* | 9.26×10^-5^ | [1] |
| *IG* production rate by T | *ϕ_81_* | s^-1^ | *ϕ_81_τT_r_IG_r_^-1^* | 2×10^-1^ | calibrated |
| *IG* production rate by M1 | *ϕ_82_* | s^-1^ | *ϕ_82_τM1_r_IG_r_^-1^* | 5×10^-2^ | calibrated |
| *IG* inhibition rate by N | *ϕ_83_* | s^-1^ | *ϕ_83_τN_r_IG_r_^-1^* | 1×10^-3^ | calibrated |

**Supplementary Table S2.** **Summary of the parameters used in the model.**

M1 production rate by IFN-γ, *φ_43_* - M2 inhibition rate by IFN-γ, *φ_53_*. The non-dimensional estimate for both parameters has been calibrated as 1 × 10^-11^, then *φ_43_* = *φ_53_* = 1.93 × 10^-18^ s^-1^.

To give the estimates of the remaining parameters, given the lack in literature of specific determinations, we adopt the method already used in [6] based on proteomic analysis, whose reliability is based on the correct reproduction of experimental data presented in [7]:

T production rate by IFN-γ, *φ_25_*. The non-dimensional estimate is 1×10^-3^, then *φ_25_* = 1.4 × 10^-10^ s^-1^.

M1 macrophages inhibition rate induced by IL-10, *φ_42_*. The non-dimensional estimate is 4×10^-4^, then *φ_42_* = 9.6 × 10^-11^ s^-1^.

M2 macrophages promotion rate induced by IL-10, *φ_51_*. The non-dimensional estimate is 2.3 ×10^-2^, then *φ_51_* = 5.53 × 10^-9^ s^-1^.

M2 macrophages inhibition rate induced by IL-6, *φ_52_*. The non-dimensional estimate is 6.5 × 10^-3^, then φ_52_ = 1.56 × 10^-9^ s^-1^.

IL-6 inhibition rate induced by IL-10, *φ_63_*. The non-dimensional estimate is 2 ×10^-3^, then *φ_63_* = 2 × 10^-7^ s^-1^.

IFN-γ production rate by T, *φ_81_*. The non-dimensional estimate is 2 ×10^-1^, then *φ_81_* = 1.4 × 10^-2^ s^-1^.

IFN-γ production rate by M1, *φ_82_*. The non-dimensional estimate is 5 ×10^-2^, then *φ_82_* = 2.6 × 10^-3^ s^-1^.

IFN-γ inhibition rate by IL-10, *φ_83_*. The non-dimensional estimate is 1 ×10^-3^, then *φ_83_* = 1.25 × 10^-7^ s^-1^.

**2. Finite element method implementation via Galerkin’s method**

In this section we provide some details about the procedure used to solve the PDEs system eqs. (3-10), carried out using the COMSOL Multiphysics ^TM^ package, allowing to benefit from several built-in functions and libraries. The solution procedure is based on the finite element method (FEM) [8], in which a PDEs system is discretized in space using the method of lines (MOL) with a mapped mesh with 17956 square elements, and then the solution procedure starts from the initial conditions imposed. To overcome difficulties arising from non-linearity of the PDEs, the Galerkin’s method, a weighted residuals method, associated to a weak formulation of the residuals of the differential equations, is used.

Considering, for simplicity, eq. (2) rewritten as

$s_{i,t}-\alpha{(s}_{i,xx}+s_{i,yy})=f_{i}(x,y,t)$, (S1)

where subscripts ‘*t*’, and ‘*xx*’ and ‘*yy*’ denote, respectively, time differentiation, and double differentiation with respect to the spatial coordinates, recalling that the diffusive flux is expressed in terms of the Fick’ law.

Suppose, also, dividing the domain ***Ω*** into *M* elements ***Ω*** *^e^* with *N* nodes, and each element has *m* nodes with *m* interpolation functions. The species concentration in the *e*^th^ element can be interpolated by its *m* local node values using *m* local shape (or interpolation) functions as

$\tilde{s}_{i}^{e}=\sum_{j=1}^{m} g_{ij}^{e}W_{j}^{e}, i=1,\ldots,8.$ (S2)

For each species *i*, the unknown coefficients *g_ij_^e^* represent the nodal solutions, and *W_j_^e^* are the interpolation functions, both associated to the *j*^th^ node of the *e*^th^ local element, while the tilde denotes an approximate solution of *s_i_* which is usually different from the exact one. The *W_j_^e^* functions, in our case, are the Lagrange quadratic shape functions for square mapped elements as provided in the COMSOL Multiphysics^TM^ package. Inserting $\tilde{s}_{i}^{e}$ into eq. (S1) we obtain the residual *R_i_^e^*, i.e., the interpolation error, in each *e*^th^ element,

${R_{i}^{e}=\tilde{s}}_{i,t}-\alpha{(\tilde{s}}_{i,xx}+\tilde{s}_{i,yy})-f_{i}, i=1,\ldots,8.$ (S3)

If *R_i_^e^* is equal to zero in each element we have the exact solution, but in practice this never happens. Instead, the use of the Galerkin’s method allows to minimize the residual in each element by weighting *R_i_^e^* with the interpolation functions *W_j_^e^*, which are the same shape functions used in eq. (S2), imposing

$\int_{\Omega_{e}} R_{i}^{e}W_{j}^{e}{d\Omega}^{e}=0, i=1,\ldots,8, j=1,\ldots,m,$ (S4)

in practice requiring the residual to be orthogonal to a set of weighting functions, from which the *m* nodal solutions *g_ij_^e^* are obtained, in each element and for each species. The time derivative of $\tilde{s}_{i}^{e}$ is computed by COMSOL Multiphysics^TM^ using the backward Euler method. The solution is obtained in COMSOL Multiphysics^TM^ by applying the assembling process typical of a FEM procedure. Further details can be found in [8,9].

|  | CAR_MERTK_ | CAR_γ_ |
| --- | --- | --- |
| *t*=10 | 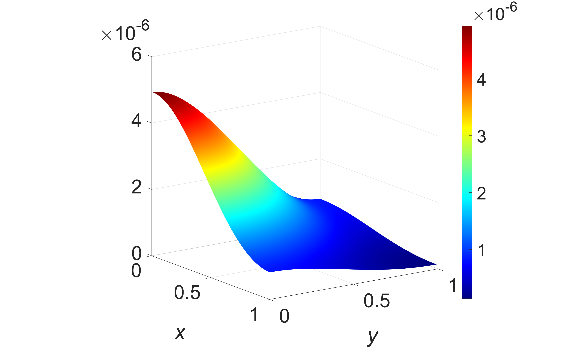 | 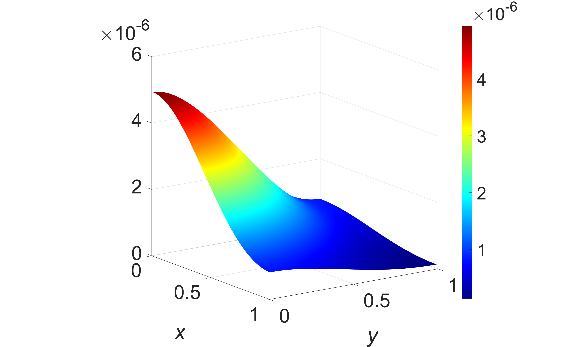 |
| *t*=20 | 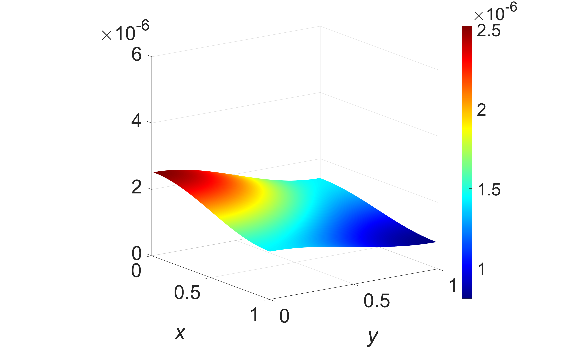 | 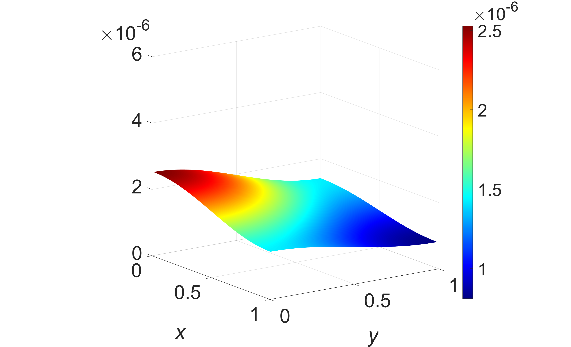 |
| *t*=30 | 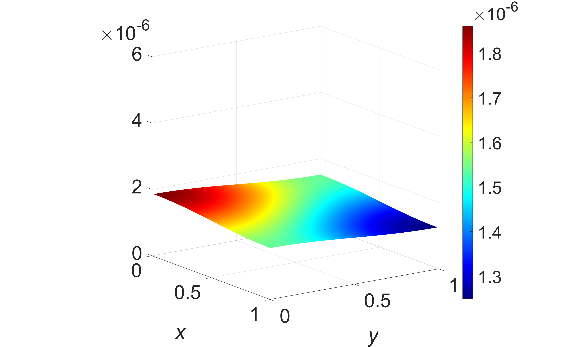 | 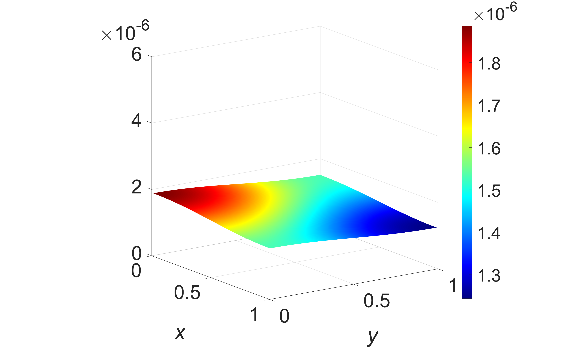 |
| *t*=40 | 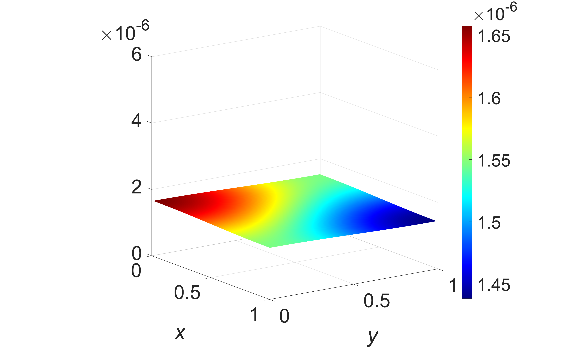 | 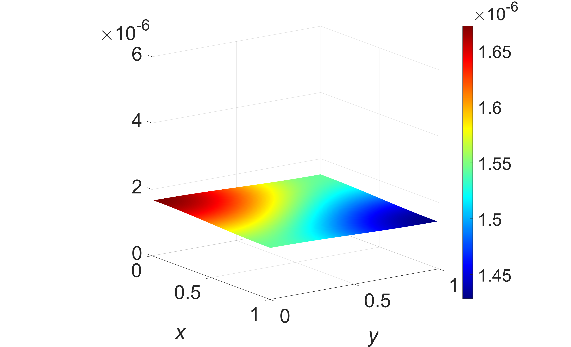 |
| *t*=50 | 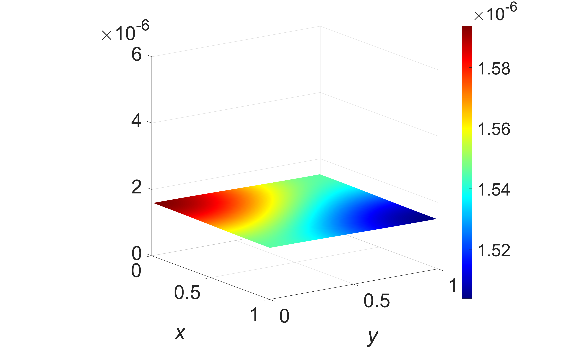 | 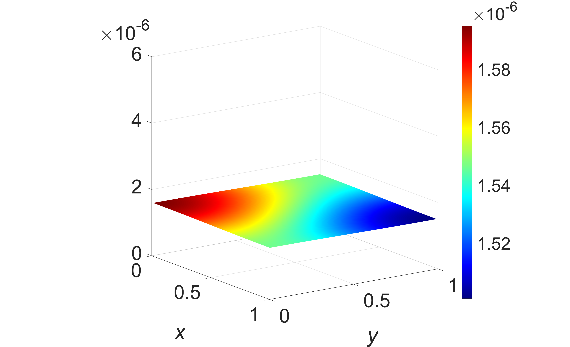 |
| *t*=60 | 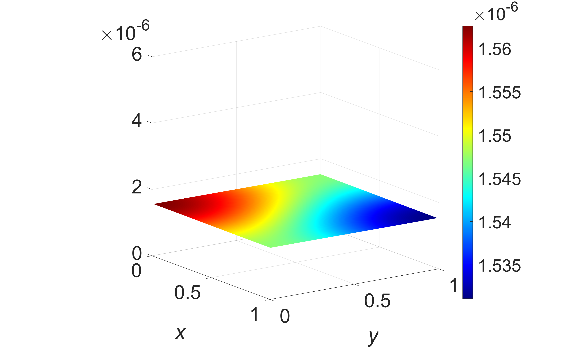 | 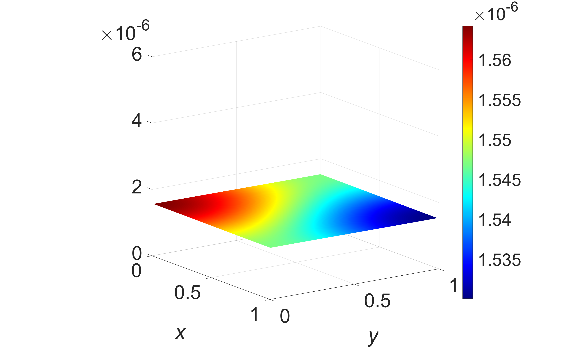 |

**Supplementary Figure S1. Surface distribution dynamics of SARS-CoV-2 virions.** The density is linearly mapped in colour scale between the blue and red colours in the [0,1]×[0,1] square domain. Starting from the top row, the variable evolution has been computed at *t* = 10 (~1.16 days), *t* = 20 (~2.3 days), *t* = 30 (~3.47 days), *t* = 40 (~4.63 days), *t* = 50 (~5.79 days) and *t* = 60 (~6.94 days), while the first and second column of panels refer to CAR_MERTK_ and CAR_γ_, respectively.

|  | CAR_MERTK_ | CAR_γ_ |
| --- | --- | --- |
| *t*=10 | 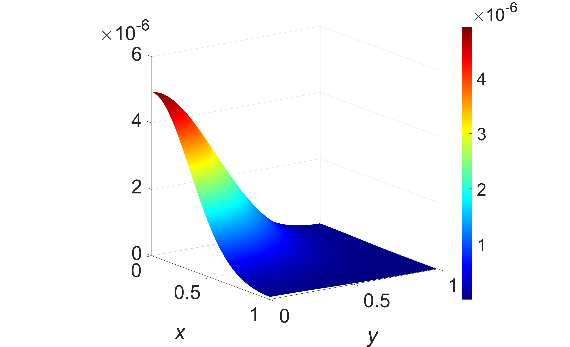 | 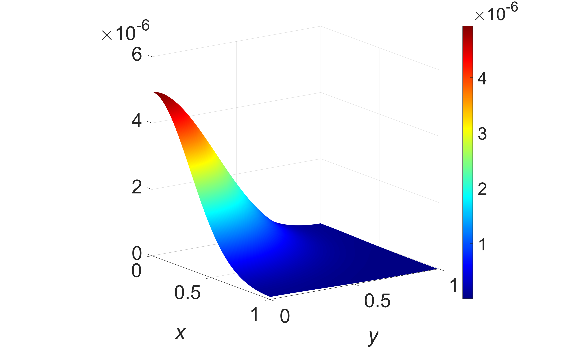 |
| *t*=20 | 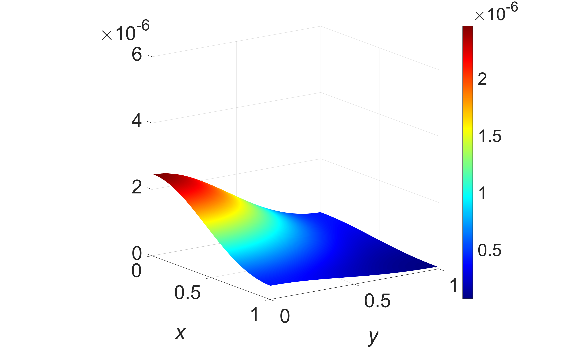 | 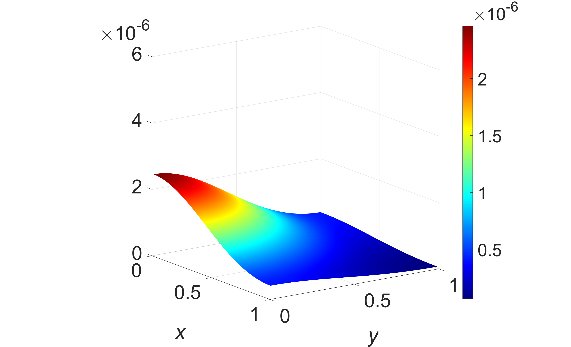 |
| *t*=30 | 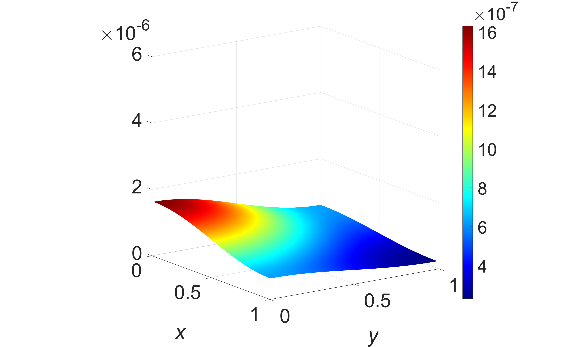 | 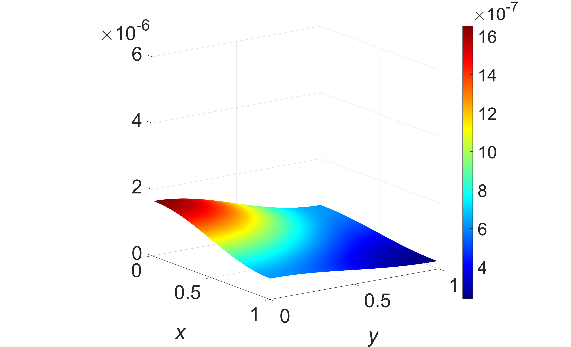 |
| *t*=40 | 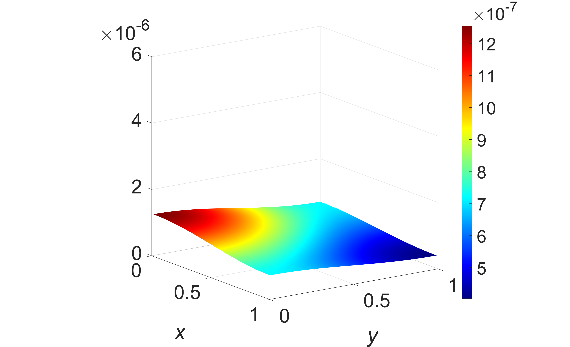 | 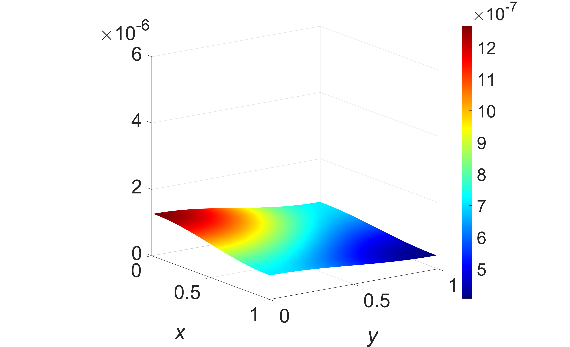 |
| *t*=50 | 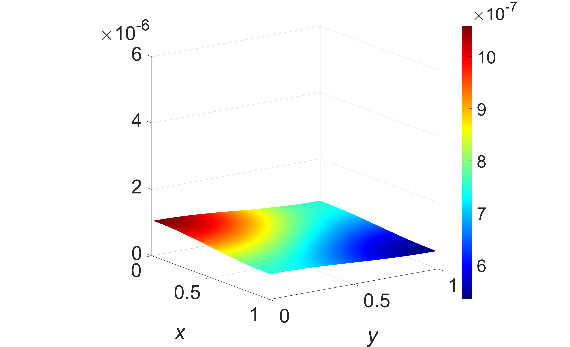 | 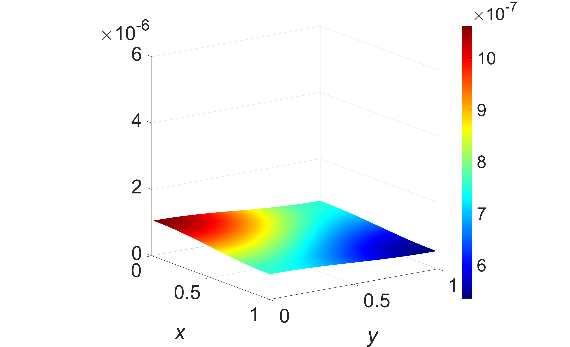 |
| *t*=60 | 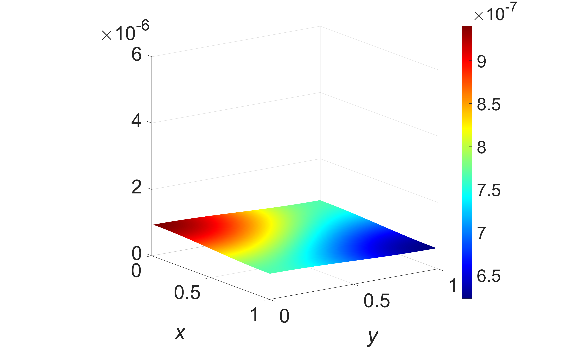 | 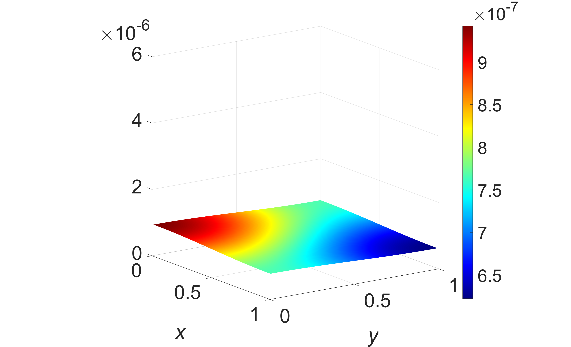 |

**Supplementary Figure S2. Surface distribution dynamics of infected CD4^+^ - T cell.** The density is linearly mapped in colour scale between the blue and red colours in the [0,1]×[0,1] square domain. Starting from the top row, the variable evolution has been computed at *t* = 10 (~1.16 days), *t* = 20 (~2.3 days), *t* = 30 (~3.47 days), *t* = 40 (~4.63 days), *t* = 50 (~5.79 days) and *t* = 60 (~6.94 days), while the first and second column of panels refer to *φ_52_* = 1 × 10^-3^ and *φ_52_* = 2 × 10^-3^, respectively.

|  | CAR_MERTK_ | CAR_γ_ |
| --- | --- | --- |
| *t*=10 | 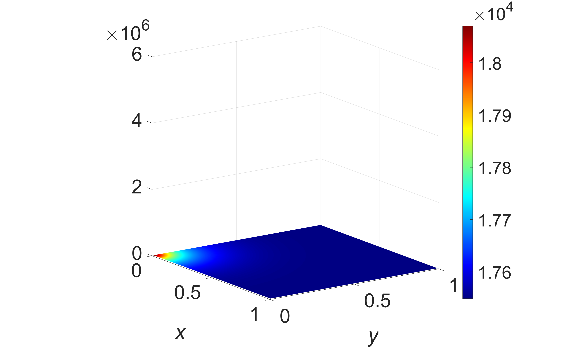 | 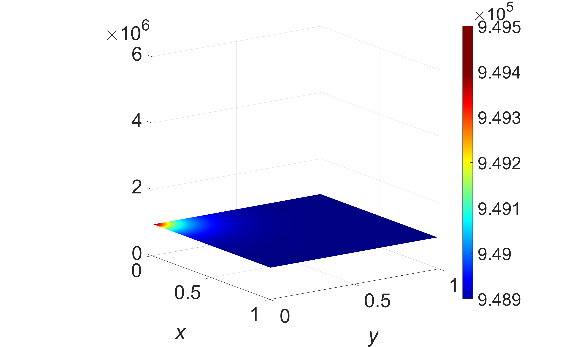 |
| *t*=20 | 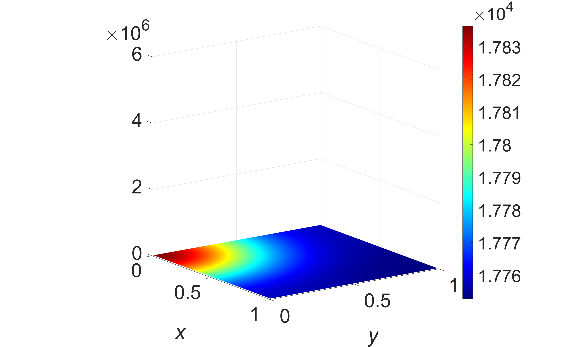 | 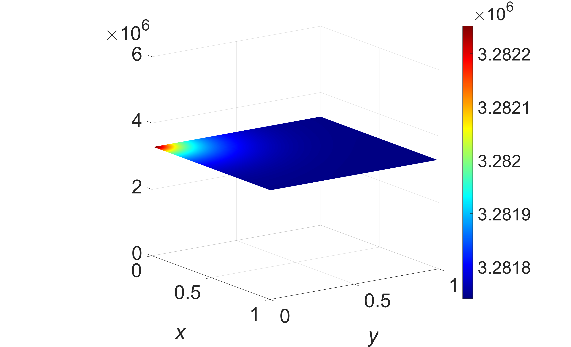 |
| *t*=30 | 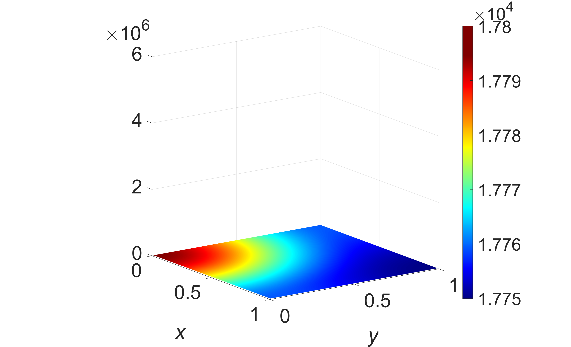 | 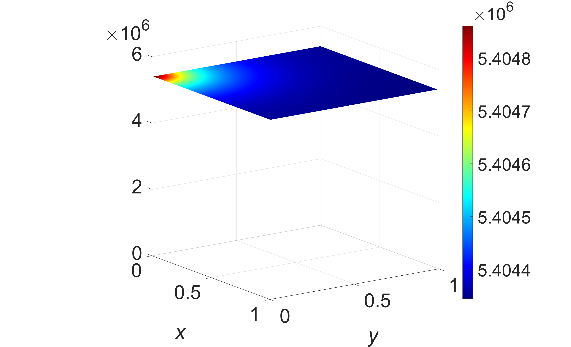 |
| *t*=40 | 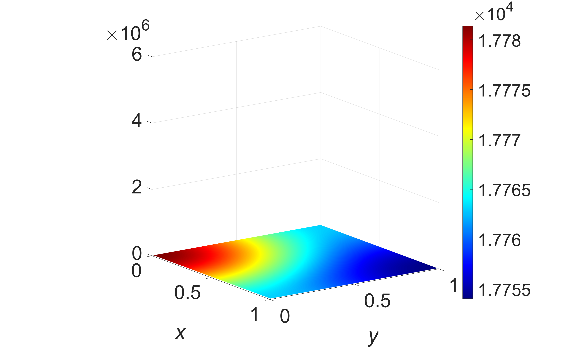 | 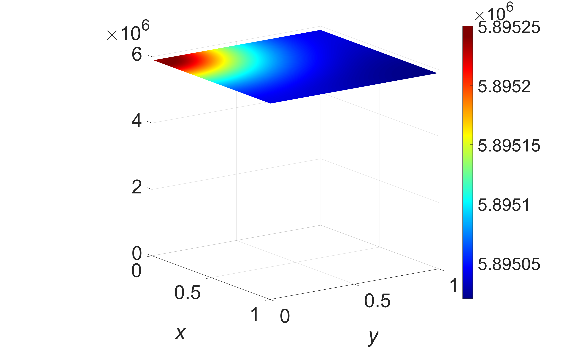 |
| *t*=50 | 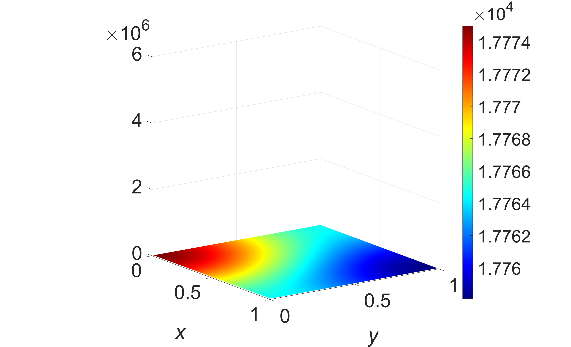 | 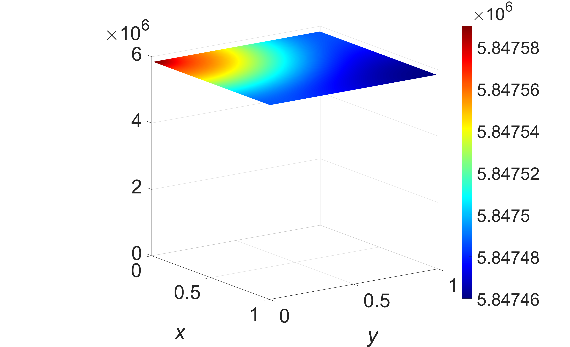 |
| *t*=60 | 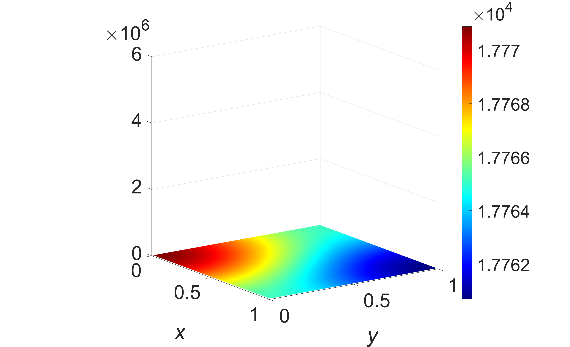 | 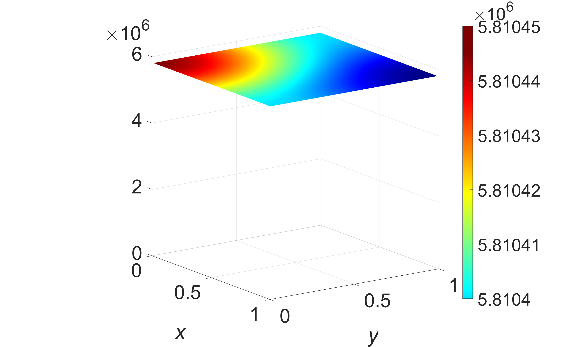 |

**Supplementary Figure S3.** **Surface distribution dynamics of CD4^+^ - T cell.** The density is linearly mapped in colour scale between the blue and red colours in the [0,1]×[0,1] square domain. Starting from the top row, the variable evolution has been computed at *t* = 10 (~1.16 days), *t* = 20 (~2.3 days), *t* = 30 (~3.47 days), *t* = 40 (~4.63 days), *t* = 50 (~5.79 days) and *t* = 60 (~6.94 days), while the first and second column of panels refer to *φ_52_* = 1 × 10^-3^ and *φ_52_* = 2 × 10^-3^, respectively.

|  | CAR_MERTK_ | CAR_γ_ |
| --- | --- | --- |
| *t*=10 | 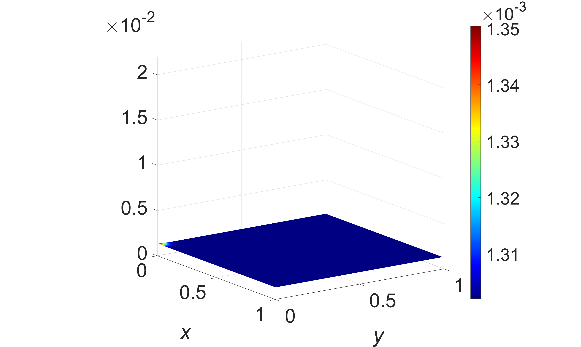 | 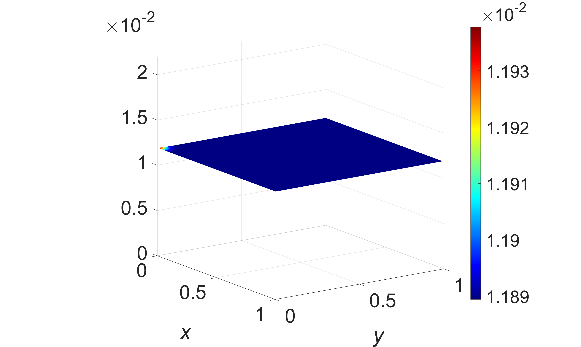 |
| *t*=20 | 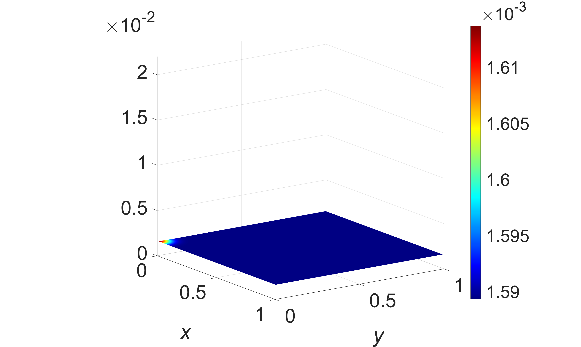 | 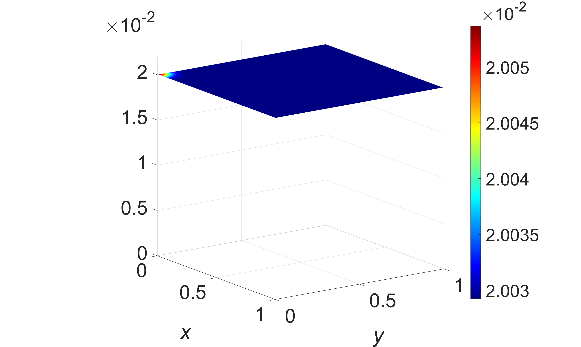 |
| *t*=30 | 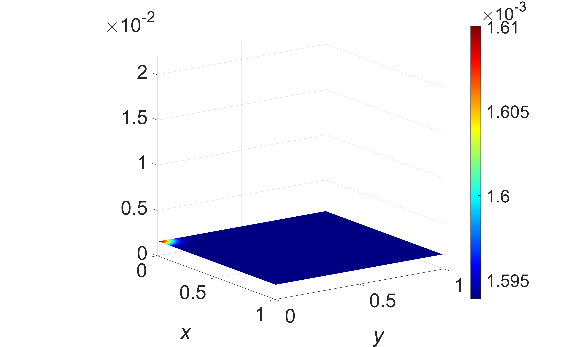 | 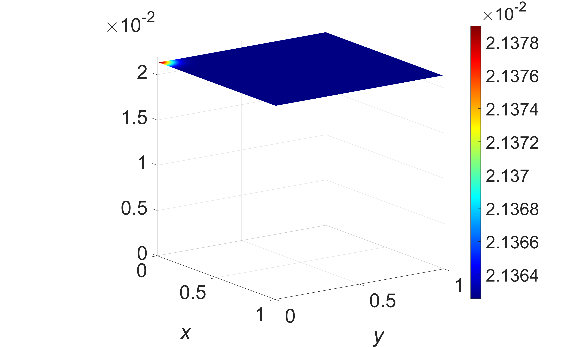 |
| *t*=40 | 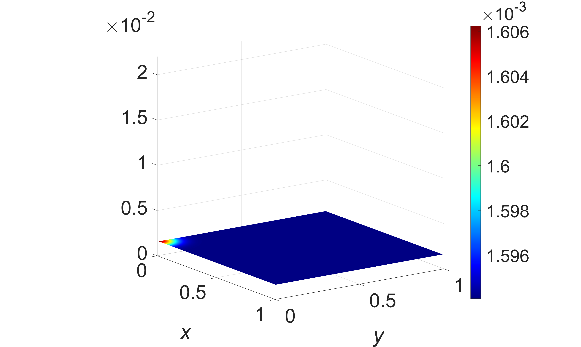 | 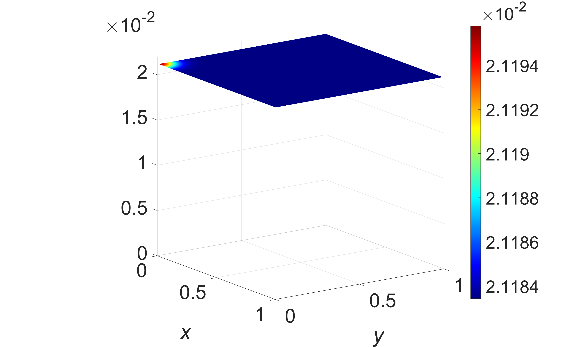 |
| *t*=50 | 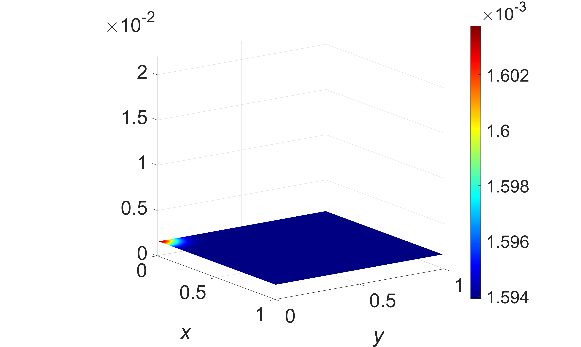 | 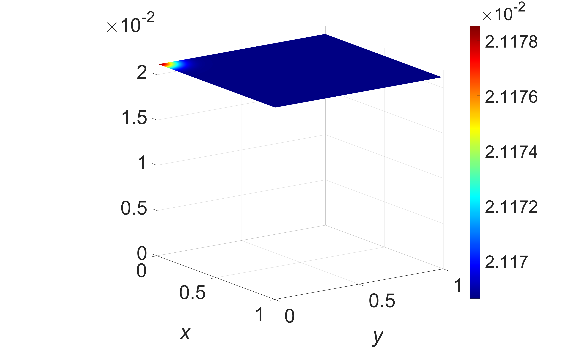 |
| *t*=60 | 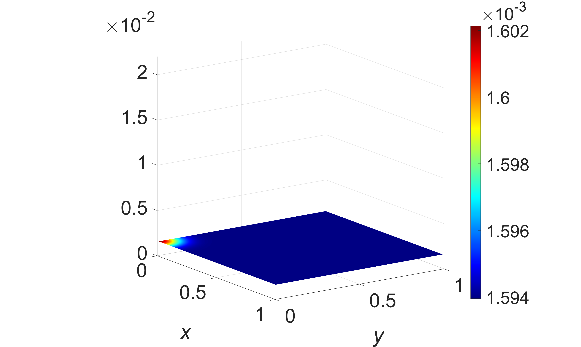 | 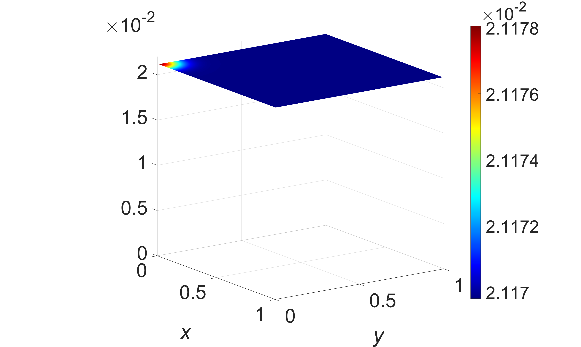 |

**Supplementary Figure S4.** **Surface distribution dynamics of M1.** The density is linearly mapped in colour scale between the blue and red colours in the [0,1]×[0,1] square domain. Starting from the top row, the variable evolution has been computed at *t* = 10 (~1.16 days), *t* = 20 (~2.3 days), *t* = 30 (~3.47 days), *t* = 40 (~4.63 days), *t* = 50 (~5.79 days) and *t* = 60 (~6.94 days), while the first and second column of panels refer to *φ_52_* = 1 × 10^-3^ and *φ_52_* = 2 × 10^-3^, respectively.

|  | CAR_MERTK_ | CAR_γ_ |
| --- | --- | --- |
| *t*=10 | 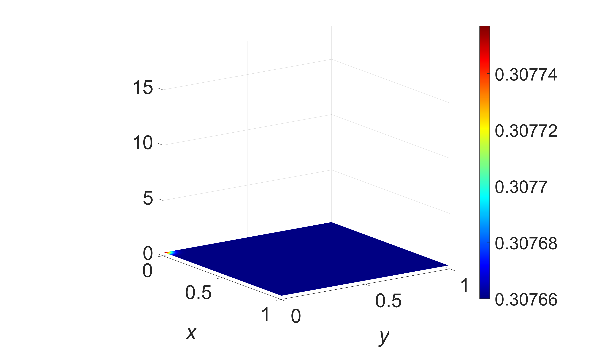 | 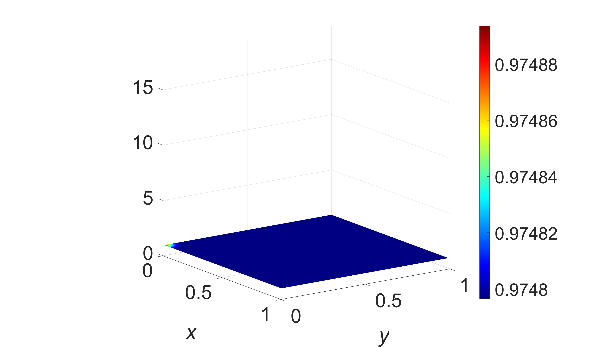 |
| *t*=20 | 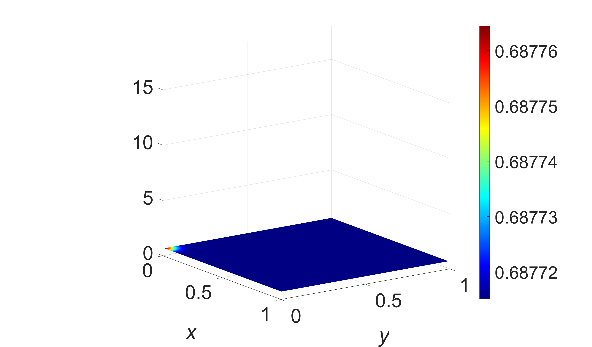 | 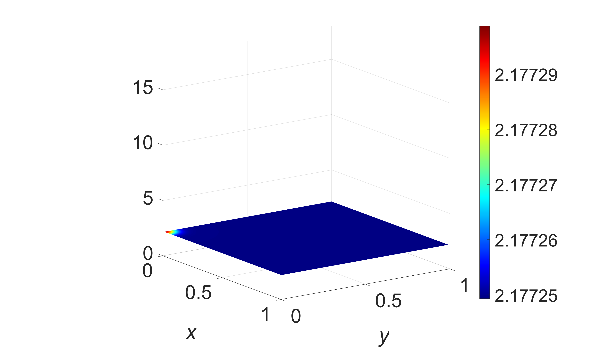 |
| *t*=30 | 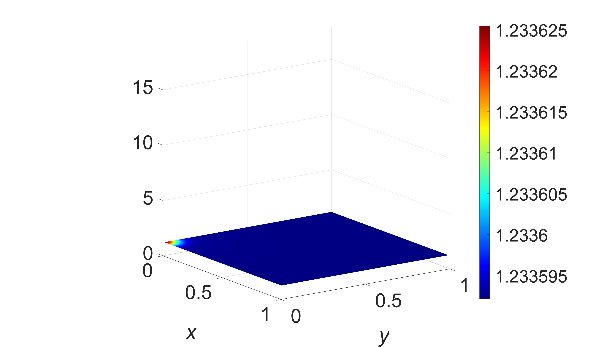 | 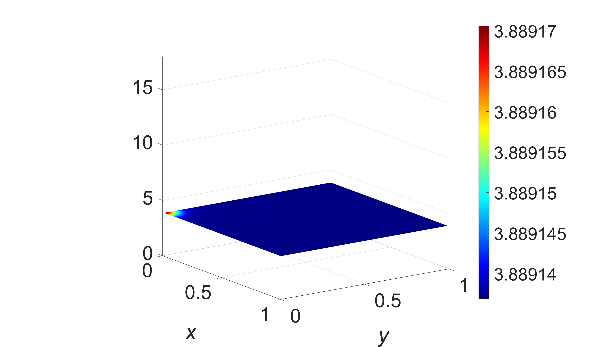 |
| *t*=40 | 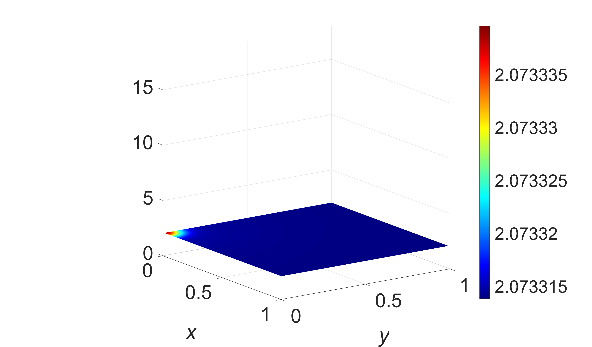 | 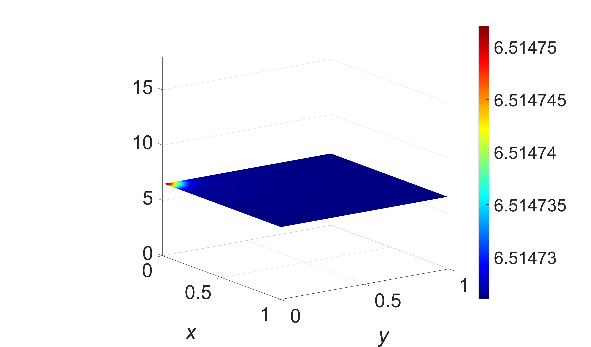 |
| *t*=50 | 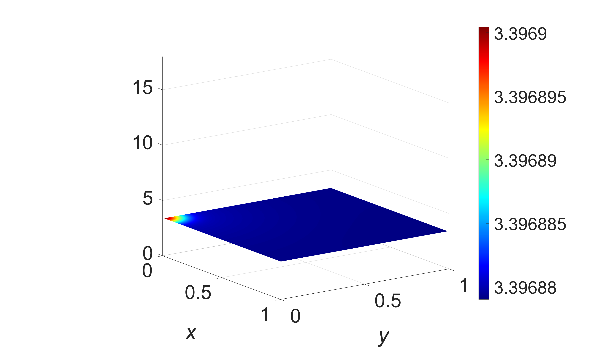 | 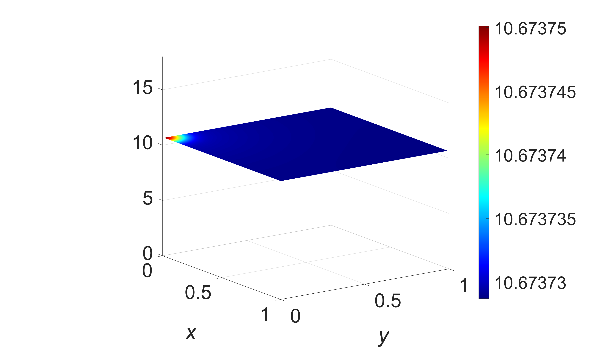 |
| *t*=60 | 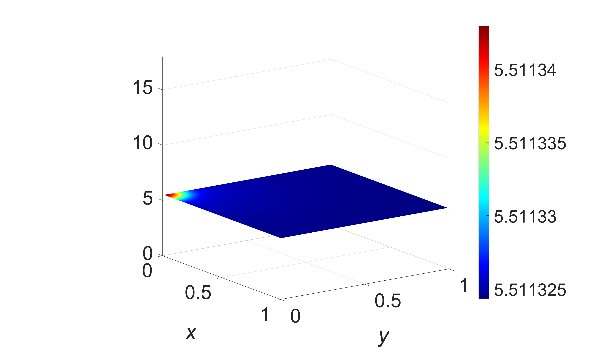 | 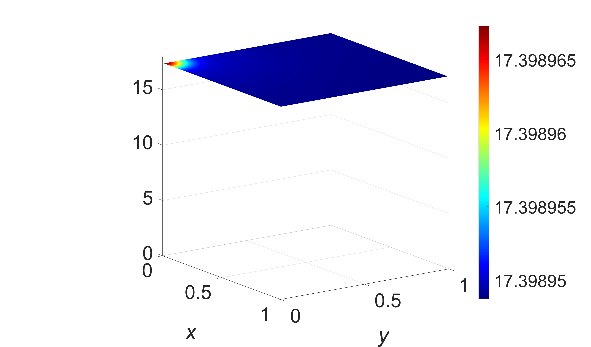 |

**Supplementary Figure S5.** **Surface distribution dynamics of M2.** The density is linearly mapped in colour scale between the blue and red colours in the [0,1]×[0,1] square domain. Starting from the top row, the variable evolution has been computed at *t* = 10 (~1.16 days), *t* = 20 (~2.3 days), *t* = 30 (~3.47 days), *t* = 40 (~4.63 days), *t* = 50 (~5.79 days) and *t* = 60 (~6.94 days), while the first and second column of panels refer to *φ_52_* = 1 × 10^-3^ and *φ_52_* = 2 × 10^-3^, respectively.

|  | CAR_MERTK_ | CAR_γ_ |
| --- | --- | --- |
| *t*=10 | 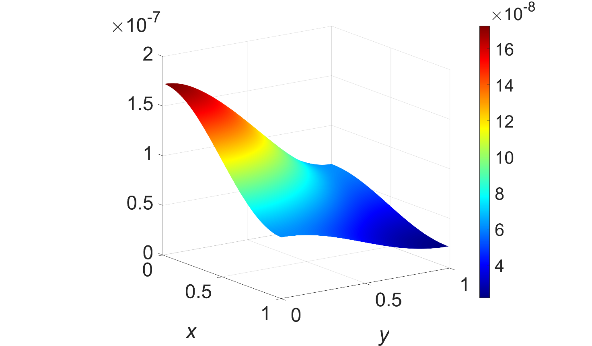 | 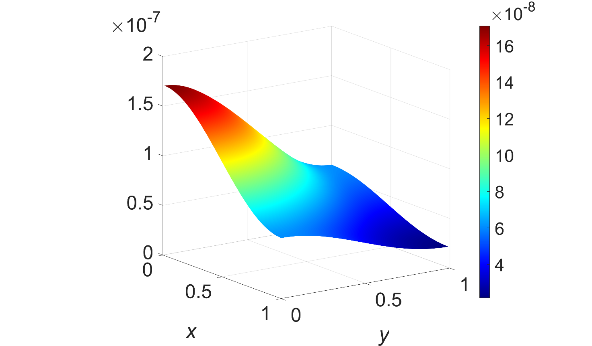 |
| *t*=20 | 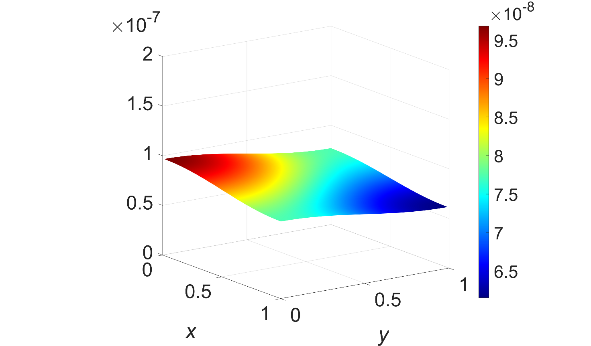 | 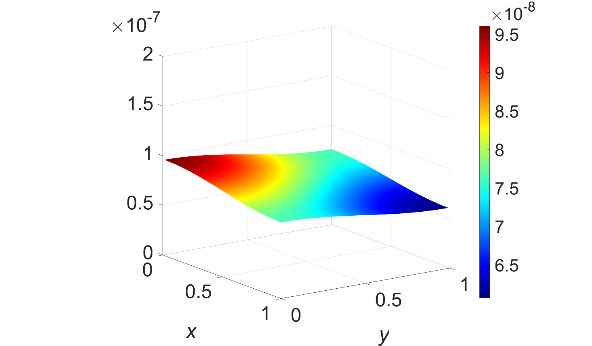 |
| *t*=30 | 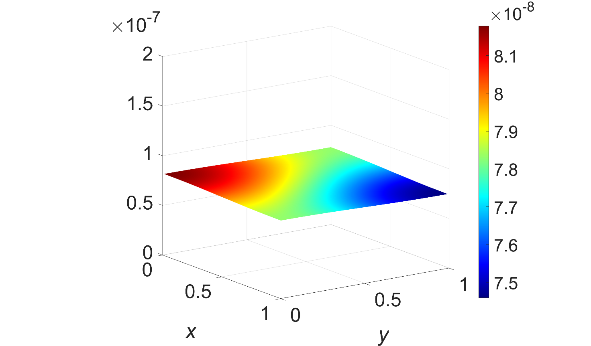 | 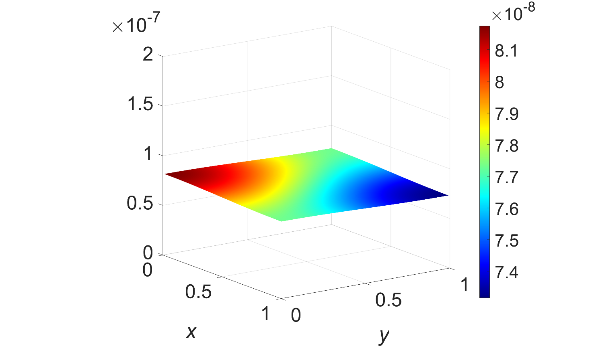 |
| *t*=40 | 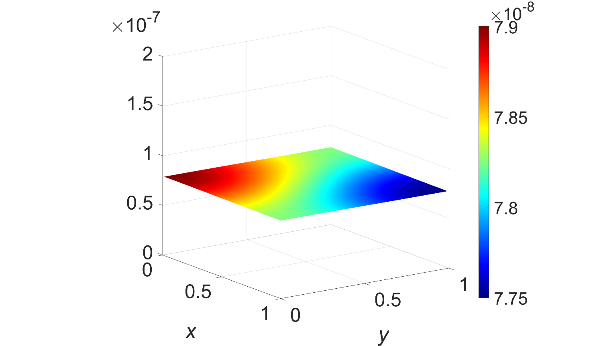 | 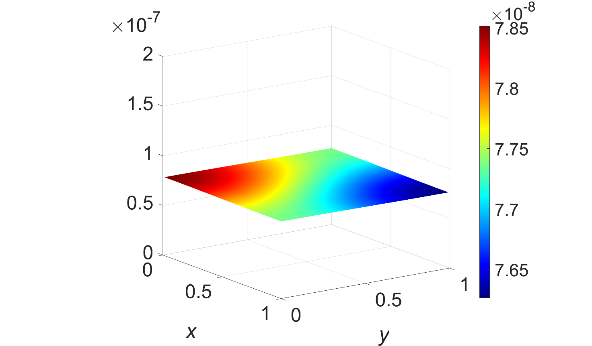 |
| *t*=50 | 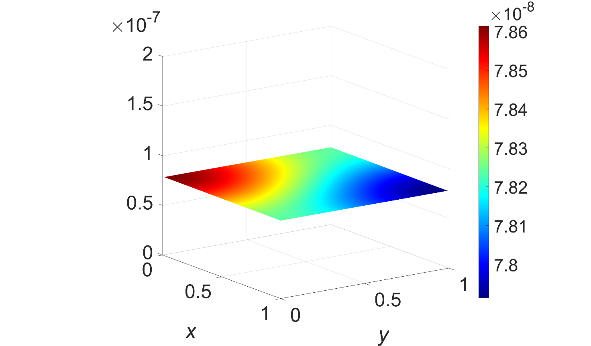 | 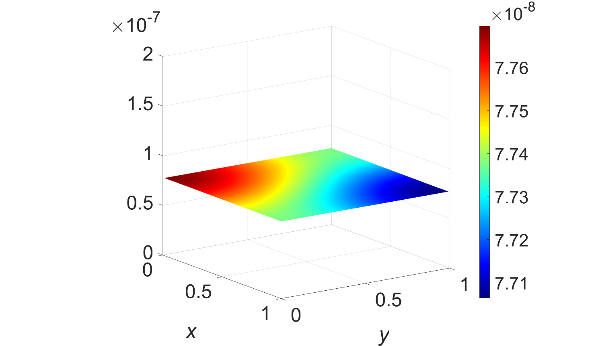 |
| *t*=60 | 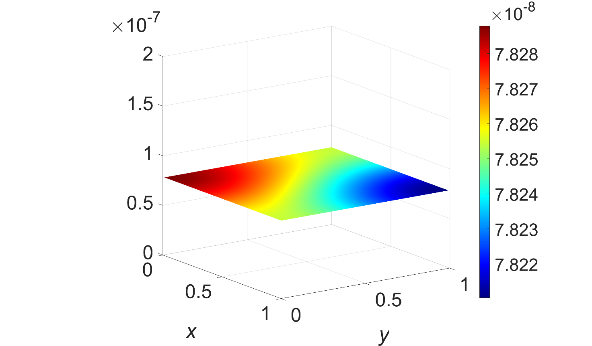 | 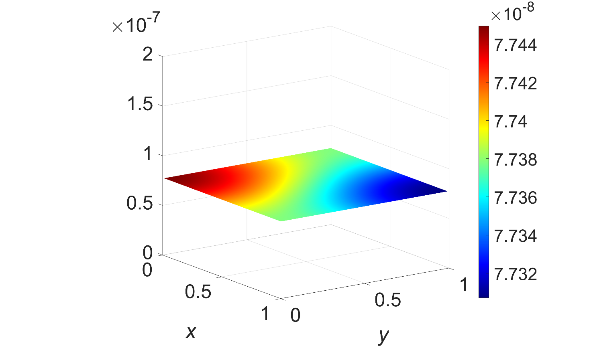 |

**Supplementary Figure S6.** **Surface distribution dynamics of IL-6.** The density is linearly mapped in colour scale between the blue and red colours in the [0,1]×[0,1] square domain. Starting from the top row, the variable evolution has been computed at *t* = 10 (~1.16 days), *t* = 20 (~2.3 days), *t* = 30 (~3.47 days), *t* = 40 (~4.63 days), *t* = 50 (~5.79 days) and *t* = 60 (~6.94 days), while the first and second column of panels refer to *φ_52_* = 1 × 10^-3^ and *φ_52_* = 2 × 10^-3^, respectively.

|  | CAR_MERTK_ | CAR_γ_ |
| --- | --- | --- |
| *t*=10 | 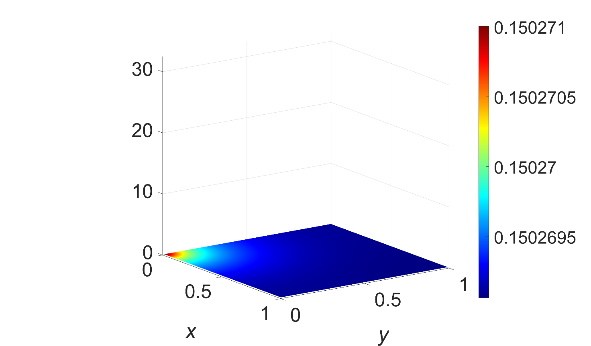 | 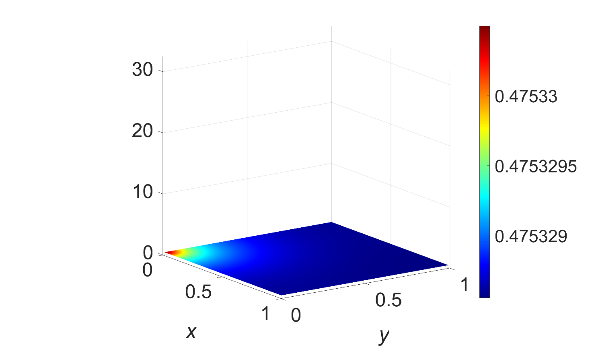 |
| *t*=20 | 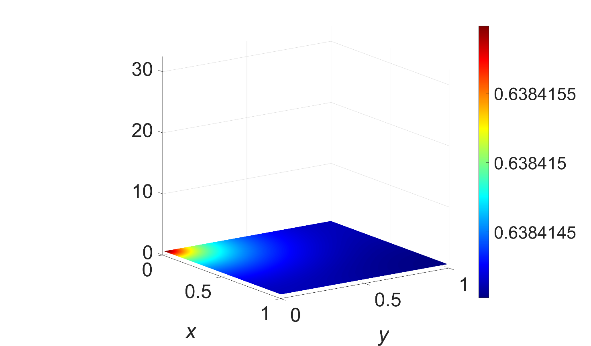 | 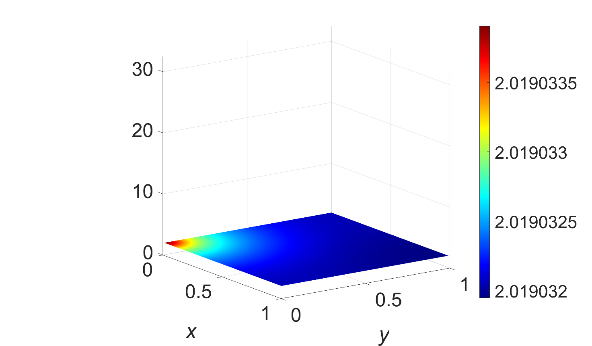 |
| *t*=30 | 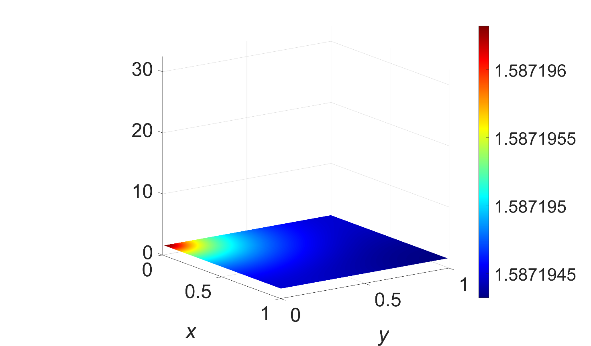 | 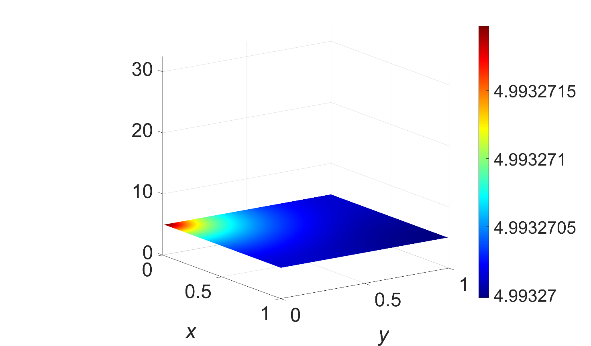 |
| *t*=40 | 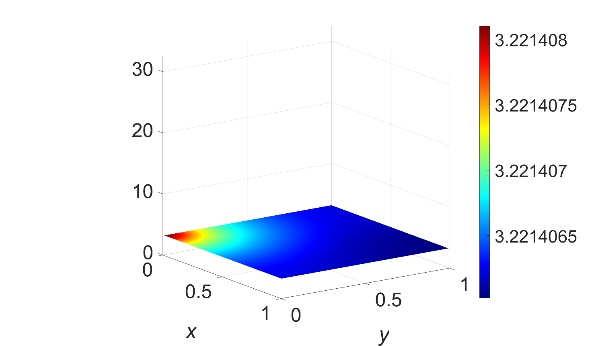 | 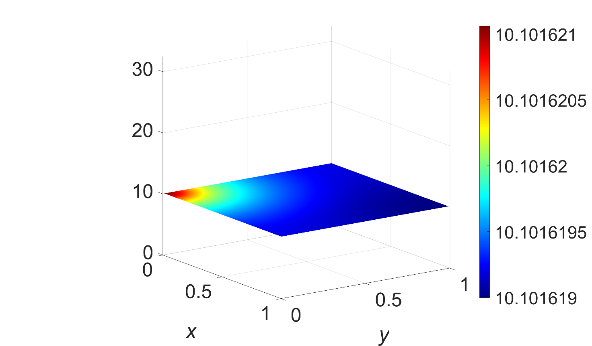 |
| *t*=50 | 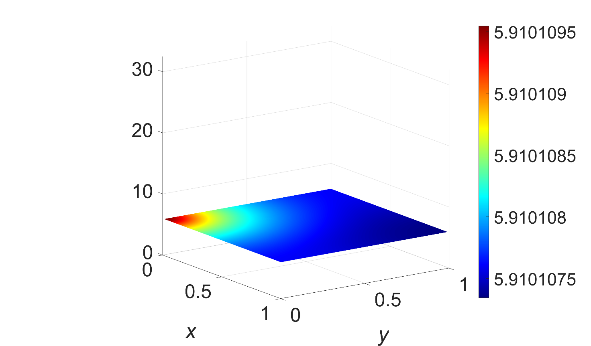 | 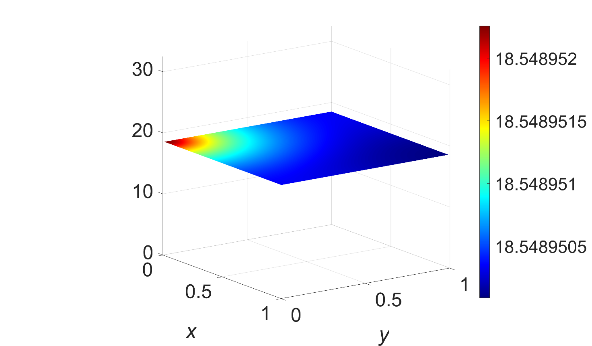 |
| *t*=60 | 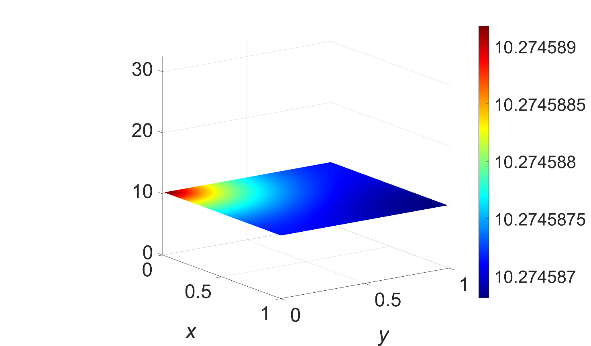 | 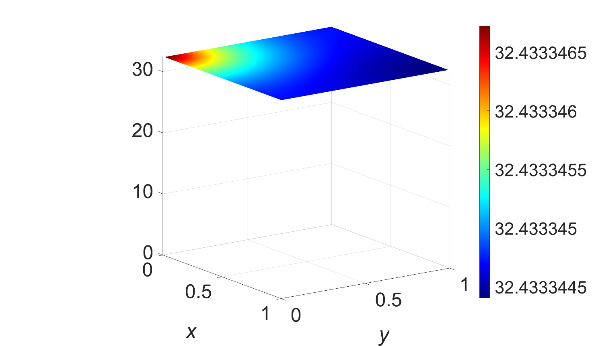 |

**Supplementary Figure S7.** **Surface distribution dynamics of IL-10.** The density is linearly mapped in colour scale between the blue and red colours in the [0,1]×[0,1] square domain. Starting from the top row, the variable evolution has been computed at *t* = 10 (~1.16 days), *t* = 20 (~2.3 days), *t* = 30 (~3.47 days), *t* = 40 (~4.63 days), *t* = 50 (~5.79 days) and *t* = 60 (~6.94 days), while the first and second column of panels refer to *φ_52_* = 1 × 10^-3^ and *φ_52_* = 2 × 10^-3^, respectively.

|  | CAR_MERTK_ | CAR_γ_ |
| --- | --- | --- |
| *t*=10 | 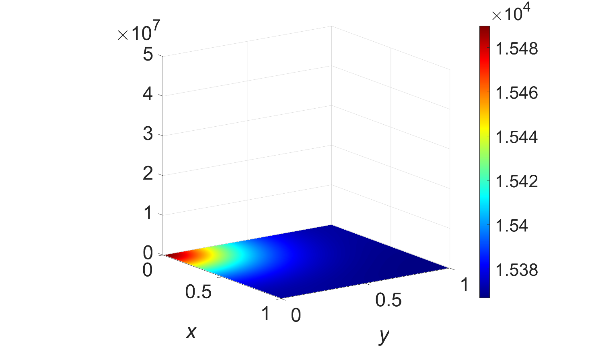 | 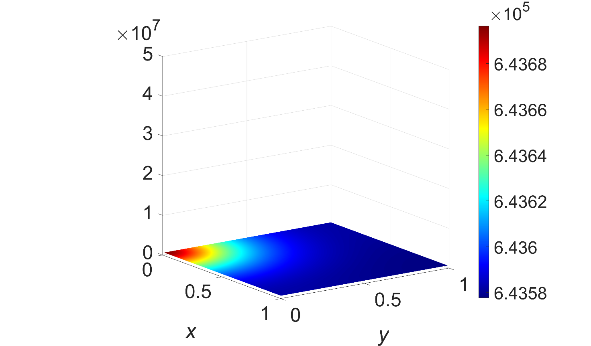 |
| *t*=20 | 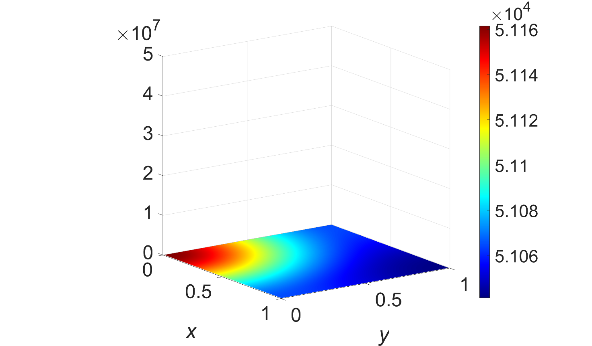 | 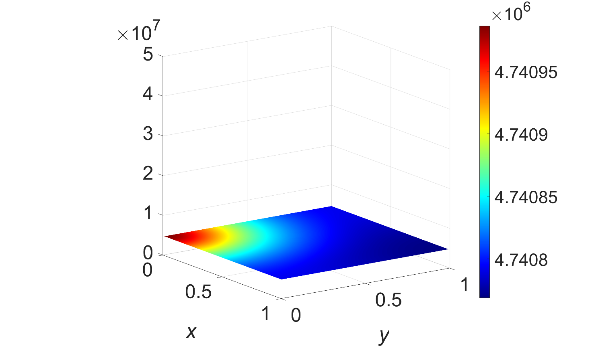 |
| *t*=30 | 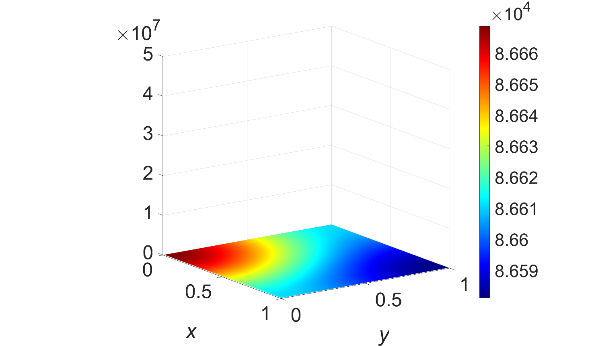 | 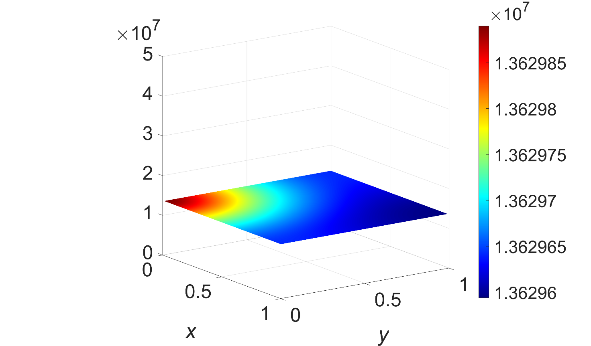 |
| *t*=40 | 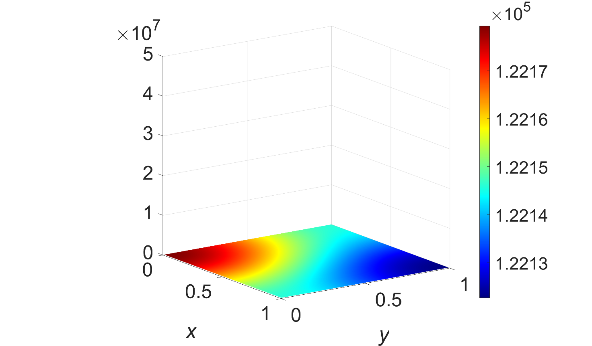 | 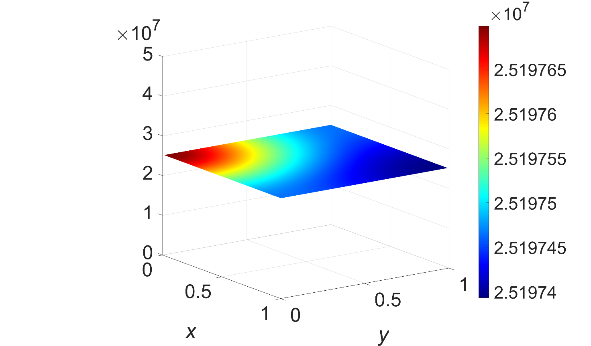 |
| *t*=50 | 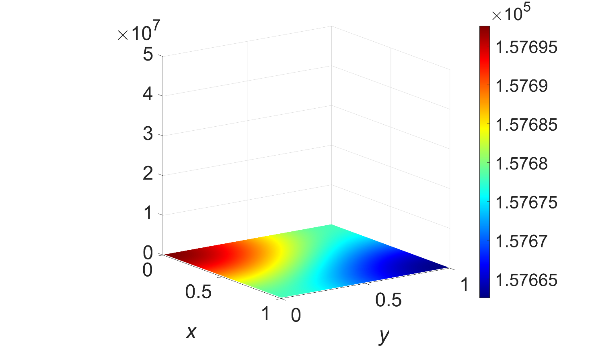 | 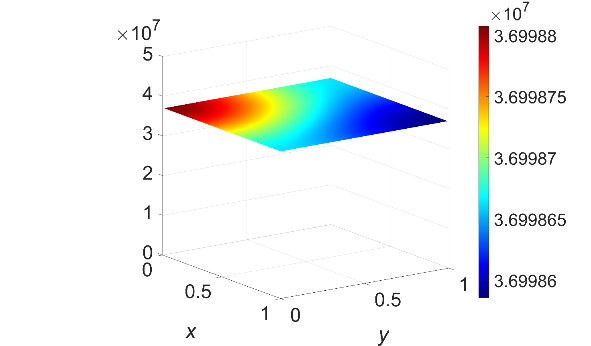 |
| *t*=60 | 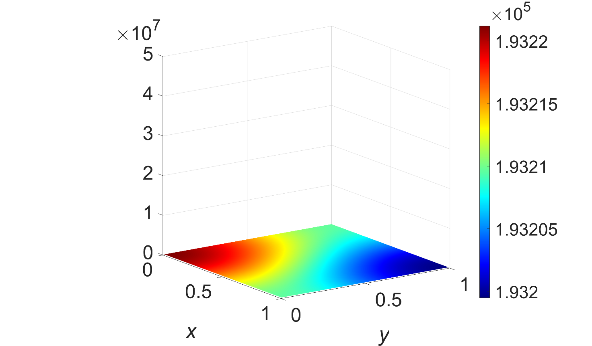 | 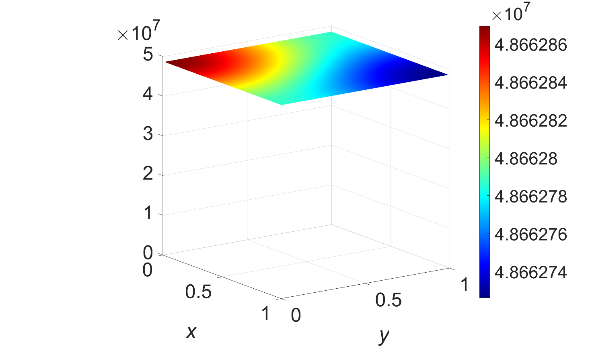 |

**Supplementary Figure S8.** **Surface distribution dynamics of IFN-γ.** The density is linearly mapped in colour scale between the blue and red colours in the [0,1]×[0,1] square domain. Starting from the top row, the variable evolution has been computed at *t* = 10 (~1.16 days), *t* = 20 (~2.3 days), *t* = 30 (~3.47 days), *t* = 40 (~4.63 days), *t* = 50 (~5.79 days) and *t* = 60 (~6.94 days), while the first and second column of panels refer to *φ_52_* = 1 × 10^-3^ and *φ_52_* = 2 × 10^-3^, respectively.

**References**

1. Amoddeo, A. A mathematical model and numerical simulation for SARS‑CoV‑2 dynamics. *Scientific Reports* **13**, 4575; https://doi.org/10.1038/s41598-023-31733-2 (2023).

2. Amoddeo, A. A moving mesh study for diffusion induced effects in avascular tumour growth. *Comput. Math. Appl.* **75,** 2508-2519; https://doi.org/10.1016/j.camwa.2017.12.024 (2018).

3. Liao, K.L., Bai, X.F. & Friedman A. Mathematical modeling of interleukin-27 induction of anti-tumor T cells response. *PLoS One* **9,** e91844; doi:10.1371/journal.pone.0091844 (2014).

4. https://www.invivogen.com/sites/default/files/invivogen/products/files/rhifn_gamma_tds.pdf

5. Marée, A.F.M., Komba, M., Finegood, D.T. & Edelstein-Keshet ,L. A quantitative comparison of rates of phagocytosis and digestion of apoptotic cells by macrophages from normal (BALB/c) and diabetes-prone (NOD) mice. *J. Appl. Physiol.* **104**, 157-169; doi:10.1152/japplphysiol.00514.2007 (2008).

6. Frieboes, H.B., Curtis, L.T., Wu, M., Kani, K. & Mallick, P. Simulation of the protein-shedding kinetics of a fully vascularized tumor. *Cancer Inform.***14,** 163–175; doi: 10.4137/CIN.S35374 (2015).

7. Fu, W. et al. CAR macrophages for SARS-CoV-2 immunotherapy. *Front. Immunol.* **12**, 669103; doi: 10.3389/fimmu.2021.669103 (2021).

8. Zienkiewicz, O.C. & Taylor, R.L. *The Finite Element Method* (Butterworth–Heinemann, Oxford, 2002).

9. Kwon, Y.W., Bang, H.C. *The Finite Element Method* Using MATLAB (CRC Press, Boca Raton, 2000).
